# Identification of the Key Role of NF-κB Signaling Pathway in the Treatment of Osteoarthritis With Bushen Zhuangjin Decoction, a Verification Based on Network Pharmacology Approach

**DOI:** 10.3389/fphar.2021.637273

**Published:** 2021-04-12

**Authors:** Yunteng Xu, Hui Li, Xiaojuan He, Yanfeng Huang, Shengjie Wang, Lili Wang, Changlong Fu, Hongzhi Ye, Xihai Li, Tetsuya Asakawa

**Affiliations:** ^1^ College of Integrative Medicine, Fujian University of Traditional Chinese Medicine, Fuzhou, China; ^2^ Academy of Integrative Medicine, Fujian University of Traditional Chinese Medicine, Fuzhou, China; ^3^ Fujian Key Laboratory of Integrative Medicine on Geriatrics, Fuzhou, China; ^4^ College of Pharmacy Science, Fujian University of Traditional Chinese Medicine, Fuzhou, China; ^5^ Research Base of Traditional Chinese Medicine Syndrome, Fujian University of Traditional Chinese Medicine, Fuzhou, China; ^6^ Department of Neurosurgery, Hamamatsu University School of Medicine, Hamamatsu-city, Japan; ^7^ Department of Neurology, The Eighth Affiliated Hospital, Sun Yat-Sen University, Guangzhou, China

**Keywords:** network pharmacology approach, complex herbal formulations, molecular targets, osteoarthritis, Bushen Zhuangjin decoction, NF-κB signaling pathway

## Abstract

This study aimed to identify whether the NF-κB signaling pathway plays a key role in the treatment of osteoarthritis (OA) with Bushen Zhuangjin Decoction (BZD) based on a typical network pharmacology approach (NPA). Four sequential experiments were performed: 1) conventional high-performance liquid chromatography (HPLC), 2) preliminary observation of the therapeutic effects of BZD, 3) NPA using three OA-related gene expression profiles, and 4) verification of the key pathway identified by NPA. Only one HPLC-verified compound (paeoniflorin) was identified from the candidate compounds discovered by NPA. The genes verified in the preliminary observation were also identified by NPA. NPA identified a key role for the NF-κB signaling pathway in the treatment of OA with BZD, which was confirmed by conventional western blot analysis. This study identified and verified NF-κB signaling pathway as the most important inflammatory signaling pathway involved in the mechanisms of BZD for treating OA by comparing the NPA results with conventional methods. Our findings also indicate that NPA is a powerful tool for exploring the molecular targets of complex herbal formulations, such as BZD.

## Introduction

Osteoarthritis (OA) is common in the aging population. It has been regarded as a severe public health concern since it remarkably reduces quality of life (Ashraf et al., 2018; Kwon et al., 2018). The mechanisms of OA are complicated and not fully understood. Many previous studies have documented the involvement of molecules and inflammatory signaling pathways in the development and progression of OA by regulation of the oxidative stress, cellular apoptosis, inflammatory response and microRNAs (Miyaki and Asahara, 2012; Mobasheri et al., 2015; Bouderlique et al., 2016; Saito and Tanaka, 2017). Of those, NF-κB signaling pathway plays a distinctive role in OA pathogenesis. Rogoglou and Papavassiliou discussed the role of NF-κB signaling pathway in OA and the potential pharmacological effects of suppression of the NF-κB signaling pathway (Rigoglou and Papavassiliou, 2013). Later, Lepetsos reported that NF-κB signaling pathway influences remodeling of the cartilage matrix, apoptosis of the chondrocytes, synovial inflammation, and the terminal chondrocyte differentiation (Lepetsos et al., 2019). Jimi et al. documented that NF-κB signaling pathway plays a key role in the regulation of the normal development and pathological destruction of cartilage (Jimi et al., 2019). Choi et al. therefore summarized that a comprehensive understanding of the roles of NF-κB signaling pathway is useful in the development of novel therapies against OA (Choi et al., 2019).

**GRAPHICAL ABSTRACT:**
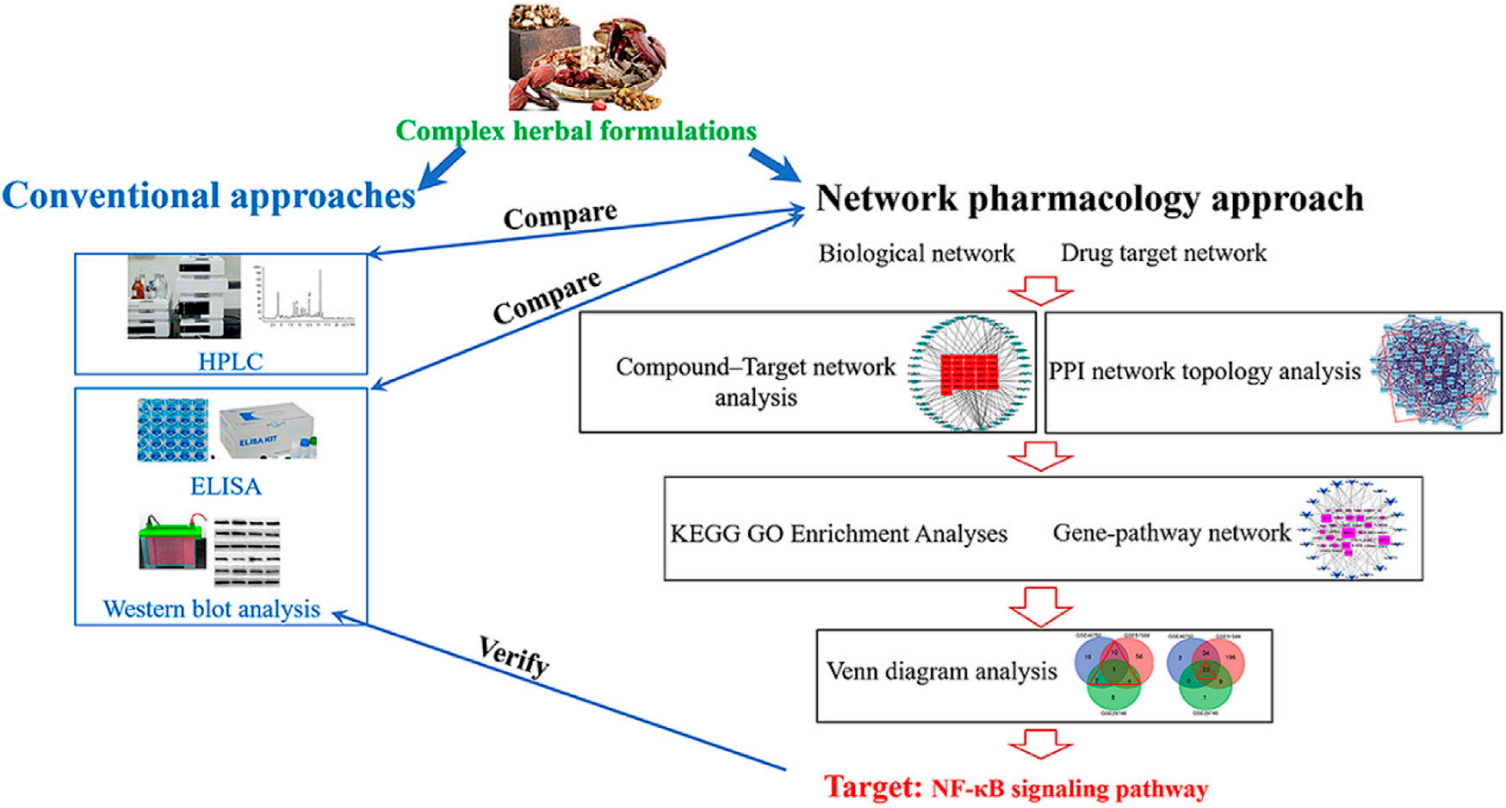


However, by far OA lacks effective therapy. Current mainstream treatments for OA, such as nonsteroidal anti-inflammatory drugs and opioids, are far from satisfactory since they can provide only temporary amelioration from symptoms (Yazici et al., 2017). It is urgent and indispensable to develop a novel and effective therapy for treating OA. In this context, many traditional Chinese medicine (TCM) treatments have been used to treat OA in China. Our laboratory carried out serials of studies to verified the efficacy of TCM medical plants for treating OA. Currently, we verified the therapeutic effects of Tougu Xiaotong capsule on tunicamycin-treated chondrocytes (Liang et al., 2019). More currently, we verified the therapeutic effects of Tougu Xiaotong capsule to treat OA *in vivo* and *in vitro*, in which the roles of p38 MAPK pathway were identidied (Li et al., 2020a; Li et al., 2020b). In addition to Tougu Xiaotong capsule, Bushen Zhuangjin Decoction (BZD) is another commonly used complex herbal formulation for treating OA in China. BZD is composed of 10 Chinese medical plants, including *Radix Rehmanniae Preparata [Rehmannia glutinosa (Gaertn.) DC.)], Radix Angelicae Sinensis [Angelica sinensis (Oliv.) Diels], Radix Dipsaci [Dipsacus asperoides C.Y.Cheng and T.M.Ai], Radix Achyranthis Bidentatae [Radix Achyranthis Bidentatae], Poria [Smilax glabra Roxb], Pericarpium Citri Reticulatae Viride [Citrus × aurantium L], Fructus Corni [Cornus officinalis Siebold and Zucc], Cortex Eucommiae [Eucommia ulmoides Oliv], Radix Paeoniae Alba [Paeonia lactiflora Pall] and Cortex Acanthopanax Radicis [Eleutherococcus nodiflorus (Dunn) S.Y.Hu]* (Supplementary Table S1)*.* BZD is widely used to treat OA in China (Huang et al., 2017). Mi observed the efficacy of BZD for treating OA in 60 patients. He found that BZD contributed to relief the OA symptoms and the total response rate was 93.3% (Mi, 2013). Guan and Zhong compared the clinical outcomes in 80 patients with OA (BZD + rehabilitation vs. rehabilitation). They found that patients treated with BZD achieved better Lysholm knee scores (Guan and Zhong, 2017). Lin et al. observed the clinical outcomes of 220 patients with OA, of those 110 cases were treated with BZD (treatment group), and 110 cases were treated with glucosamine hydrochloride (control group). They found that the BZD group had better efficacy in alleviation of pain and amelioration of the Index of Severity for OA in comparison with the control group after 12-week treatments (Lin et al., 2017). A number of Chinese literatures reported the mechanisms of BZD are associated with suppressing the apoptosis of chondrocytes (Zhou et al., 2012), promoting the proliferation of the chondrocytes (Li Y. et al., 2020), protecting the injury of chondrocytes via upregulation of Sox 9 protein (Wu et al., 2020), downregulating the expression of ROCK, Cofilin,Phospho-Cofilin,LIMK1, and Phospho-LIMK1 proteins, which were relevant to damage of the cartilage in OA (Liang et al., 2015), decreasing the serum levels of tumor necrosis factor alpha (TNF-α) and Interleukin (IL)-6 in OA patients (Zhang, 2006) and animal models (Yang et al., 2020), and increasing the activity of the superoxidase dismutase in OA rabbits (Yao et al., 2005). Our laboratory also found that BZD presented the effects of suppressing the expression of the IL-1β, TNF-α and MMP-3 in synovial fluid in rat OA models (Li et al., 2014). However, literatures in English are limited. Our previous studies have found that BZD promotes the proliferation of chondrocytes by stimulating the cell cycle (Li et al., 2015) and suppresses endoplasmic reticulum stress-mediated apoptosis in an OA cell model (Lin et al., 2015). Evidence obtained from these literatures strongly implies that anti-inflammatory mechanism may play a role in the mechanisms of BZD. Due to the key roles of NF-κB signaling pathway in the inflammatory mechanisms of OA, we therefore speculated that NF-κB signaling pathway also plays a role in the mechanisms of BZD in treating OA. Nevertheless, by far, no study elucidates the role of NF-κB signaling pathway in BZD, which is desired to be further investigated.

A novel approach, known as the network pharmacology approach (NPA) has recently attracted interest. NPA combines systematic bioactive analysis and pharmacology. An NPA simultaneously searches for molecular targets from the ingredients of a complex herbal formulation and the target disease and identifies the therapeutic targets by determining the intersections shared by the formulation and disease. This approach is believed to be useful for elucidating the synergistic effects and interactions among compounds. It also identifies potential mechanisms of multi-component and multi-target drugs using the compound–compound, compound–target, and target–disease networks (Liu et al., 2016). NPA’s usefulness for exploring putative molecular targets and mechanisms of complex herbal formulation has been widely proved.

In the present study, we used a standard NPA to identify the underlying mechanisms of BZD’s effectiveness in treating OA. In terms of the key role of NF-κB signaling pathway in OA, we hypothesized that the NF-κB signaling pathway also plays a key role in the mechanisms of BZD for treating OA. To test this hypothesis, four sequential experiments were designed: 1) detection of the chemical components, 2) preliminary verification of BZD’s effects in lipopolysaccharide (LPS)-induced OA cell model, 3) NPA for BZD, and 4) validation of the pathway(s) identified by NPA. We attempted to identify the role of NF-κB signaling pathway in the treatment of OA with BZD by comparing the NPA results with conventional methods. Moreover, the findings of this study will prove that NPA is a robust tool to identify the molecular targets of a commonly used complex herbal formulation.

## Materials and Methods

### Experiment 1. Quality Control of Bushen Zhuangjin Decoction

#### Preparation of Bushen Zhuangjin Decoction

BZD medical plants, obtained from the Third People’s Hospital, affiliated with Fujian University of TCM (Fuzhou, China), were crushed and passed through a 20–40 mesh sieve. To establish the correct BZD ratio (Supplementary Table S1), we filtered 105 g of herbal powder using 840 ml of 67% ethanol and extracted by reflux. The filtrate was evaporated using a rotary evaporator (RE-2000; Shanghai Yarong Biochemistry Instrument Factory, Shanghai, China) and then dried to a constant weight in a vacuum drying oven (DZF-300; Shanghai Yiheng Scientific Instrument Co., Shanghai, China). BZD was dissolved in phosphate-buffered saline (PBS, HyClone Laboratories, Inc., Logan, UT, United States) to a stock concentration of 40 mg/ml and stored at −80°C. The working concentration of BZD was prepared by diluting the stock solution in PBS, filtering through a 0.22 µm filter, and storing at 4°C.

#### Quality Control of Bushen Zhuangjin Decoction

BZD extracts were analyzed by HPLC using an Agilent 1200 HPLC system (Agilent, Santa Clara, CA, United States) with an Agilent 5 TC-C_18_ (250*4.6 mm) column (Supplementary Figure S1). The analytical conditions included acetonitrile (A) and 0.2% phosphoric acid in water (B) as a mobile phase, a detection wavelength of 230 nm for morroniside (purity 98%, (Supplementary Figure S1E) and paeoniflorin (purity 98%, (Supplementary Figure S1F), a detection wavelength of 212 nm for asperosaponin VI (purity 98%, (Supplementary Figure S1G) (China Institute of Food and Drug test, Beijing, China), a flow rate of 0.8 ml/min, and a column temperature of 30°C.

### Experiment 2. Preliminary Studies of the Therapeutic Effects of Bushen Zhuangjin Decoction

#### Animals

Four-week-old male Sprague Dawley rats (BW: 90–120 g, *n* = 24) were purchased from the Shanghai Slack Laboratory Animal Co. (Shanghai, China). Rats were housed in the animal center at 60% humidity, 23°C room temperature, 12-h light/dark cycle (8:00 AM–8:00 PM), with freely available food and water. Animals were treated following the National Institute of Health Guidelines for the Care and Use of Laboratory Animals. All experiments were approved and supervised by the Animal Care and Use Committee of the Fujian University of TCM (Approval number: 2020015).

#### Preparation of Chondrocytes to Establish an LPS-Induced Model

Chondrocytes were obtained and used to generate an LPS-induced cell model, as described previously (Liang et al., 2019; Li et al., 2020a; Li et al., 2020b). Briefly, chondrocytes were obtained from the knee joints of four rats at a time (six times in total). Cells were identified by Collagen II immunohistochemistry. Chondrocytes were exposed to 10 ng/ml LPS (Sigma-Aldrich, United States) for 8 h to establish the cell model (Li et al., 2020b).

#### Measuring Matrix Metallopeptidase (MMP)-9 and Interleukin (IL)-6 Levels in Cell Supernatants Treated With Bushen Zhuangjin Decoction

Chondrocytes were treated with 12.5 μg/ml, 25 μg/ml, 50 μg/ml, 100 μg/ml, 200 μg/ml BZD and 10 ng/ml LPS for 8 h. MMP-9 and IL-6 levels in the cell supernatants were measured using a standard Enzyme-Linked Immunosorbent Assay as per the manufacturer’s instructions (R&D Systems, United States). Samples (50 µL) of the cell supernatants were analyzed using an enzyme labeling instrument (model ELx800; BioTek, Winooski, VT, United States) at a wavelength of 450 nm.

#### Verification Efficacy of Bushen Zhuangjin Decoction by Western Blot Analysis

Chondrocytes were divided into a control group, a model group (LPS 10 ng/ml), and a BZD group (BZD 50 µg/ml + LPS 10 ng/ml) and incubated for 8 h. Total proteins were collected immediately using lysis buffer (Beyotime Institute of Biotechnology, Haimen, China), stored for 30 min on ice, and quantified using the bicinchoninic acid assay. A total of 20 µg protein was separated on 10% SDS-PAGE gels and transferred onto polyvinylidene fluoride membranes (Sigma-Aldrich, United States). The membranes were blocked with 5% non-fat milk and incubated with primary antibodies against tumor necrosis factor (TNF)-α (ab11564, abcam, United States), IL-1β (ab205924, abcam, United States), MyD88 (ab2064, abcam, United States), matrix metallopeptidase (MMP)-3 (ab52915, abcam, United States), TLR4 (ab217274, abcam, United States), NF-κB p65 (ab16502, abcam, United States), and GAPDH (5174s, Cell Signaling Technology, United States) overnight at 4°C. Goat anti-rabbit horseradish peroxidase-conjugated secondary antibody IgG (bs-0295G-HRP, Bioss, China) or goat anti-mouse horseradish peroxidase-conjugated secondary antibody IgG (bs-0296G-HRP, Bioss, China) was added to the membranes at room temperature. The immunocomplexes were visualized by the enhanced chemiluminescence method. The bands were quantified by scanning densitometry (Molecular Imager ChemiDoc X-Ray Spectroscopy System, cat. no. 170-8070; Bio-Rad). The blots were analyzed using Image Lab software with GAPDH as a control.

### Experiment 3. Screening of Bioactive Components and Molecular Targets of Bushen Zhuangjin Decoction Using NPA

#### Screening of Bioactive Components and Molecular Targets of Bushen Zhuangjin Decoction

All BZD components were searched using the TCM systems pharmacology database and analysis platform (TCMSP, https://tcmspw.com/tcmsp.php) (Ru et al., 2014) as well as the SymMap (https://www.symmap.org/) (Wu Y. et al., 2019). Oral bioavailability (OB ≥ 30%) and drug-like (DL ≥ 0.18), which are commonly used screening methods for the chemical composition of TCM, were chosen as screening parameters. The OB value refers to the relative amount absorbed into the systemic blood circulation after the drug is administered by an extravascular route. DL refers to the similarity of a compound with a known drug, and the class of compounds having the potential to become drugs. One hundred and thirteen eligible compounds were obtained, two for RRP, two for RAS, eight for RD, 20 for RAB, 15 for P, five for PCRV, 20 for FC, 28 for CE, and 13 for RPA.

#### Predicting the Molecular Targets for OA

Differentially expressed genes identified for OA patients were obtained from the Gene Expression Omnibus database (GEO, https://www.ncbi.nlm.nih.Gov/geo/) (Clough and Barrett, 2016). The series GSE46750 (https://www.ncbi.nlm.nih.gov/geo/query/acc.cgi?acc=GSE46750) (Lin et al., 2018) was selected to compare the gene expression profiles of inflammatory I and normal/reactive (N/R) synoviocytes from the synovium of the same OA patient. Differential expression patterns were identified in two regions of the synovium in 12 patients undergoing total knee arthroplasty. The series GSE51588 (https://www.ncbi.nlm.nih.gov/geo/quer y/acc.cgi?acc=GSE51588) (Gu et al., 2019) was also selected, which represents total RNA isolated from human OA (*n* = 20) and non-OA (*n* = 5) lateral and medial tibial plateaus of the knee. This profile was obtained by performing whole-genome microarray profiling of human osteoarthritic subchondral bone. The series GSE29746 (https://www.ncbi.nlm.nih.gov/geo/query/acc.cgi?acc= GSE29746) (Li et al., 2016) was selected to compare the gene expression profiles of OA synovial tissues with normal synovial fibroblasts from healthy individuals. The samples were derived from 11 healthy adult donors and 11 sex- and age-matched patients with OA. The original file was processed by a robust multiarray average algorithm with normalization of matrix data, and the relevant data were filtered using the Limma package to analyze the chip data twice, combining the *p*-value, and the difference multiple. The screening conditions for significantly differentially expressed genes were *p* < 0.05 with a |log 2 (fold change)| > 0.05.

#### Construction of an “Active Component-Target Network” for Bushen Zhuangjin Decoction

The compound-target network for BZD was constructed and visualized using Cytoscape 3.7.2 software. Protein–protein interaction (PPI) data were obtained from the Database of Interacting Proteins (DIP™) (Xenarios et al., 2002), Biological General Repository for Interaction Datasets (Stark et al., 2006), Human Protein Reference Database (Goel et al., 2012), IntAct Molecular Interaction Database (IntAct) (Kerrien et al., 2012), Molecular INTeraction database (MINT) (Licata et al., 2012), and the biomolecular interaction network database (Alfarano et al., 2005) using the plugin Bisogenet of Cytoscape 3.7.2 software. The PPI networks for BZD putative targets and OA-related targets were visualized with Cytoscape software.

#### Construction of Protein–Protein Interaction Networks and Screening of Key Targets

The protein–protein interaction networks for BZD and OA targets were drawn with the Biogenet plugin, and the intersection of the two networks was obtained by Cytoscape, which is the direct or indirect target regulatory network for BZD in OA. Using the network topology analysis plugin CytoNCA and filtering with Degree Centrality (DC), Betweenness Centrality (BC), Closeness Centrality (CCT), Eigenvector Centrality (EC), Local average connectivity-based method (LAC), and Network Centrality (NC), key genes were identified in the PPI network, and the core target was determined for BZD activity against OA.

#### Gene Ontology and Kyoto Encyclopedia of Genes and Genomes (KEGG) Analysis

The Gene Ontology database (GO, http://geneontology.org/) (Pomaznoy et al., 2018), which contains a molecular function (MF), biological process (BP), and cellular component (CC) data, was used to identify biological mechanisms from high-throughput genomic or transcriptome data. The functional categories were enriched within genes (false discovery rate [FDR] < 0.05), and the top 20 GO functional categories were selected. The KEGG, https://www.kegg.jp/) database (Kanehisa, 2002) was used to identify the function and biological correlation of candidate target genes. Cluster Profiler R package was used to visualize the GO and KEGG pathway data. The pathways which exhibited significant changes with an FDR < 0.05 were selected for further analysis. The genes that represented significantly regulated pathways were selected for gene-pathway network analysis. The gene-pathway network was constructed to screen key target genes involved in BZD activity.

#### Venn Diagram Analysis

A Venn diagram analysis was performed to identify the key target genes by finding intersections among the GSE46750, GSE51588 and GSE297 profiles. The results of the target gene identification for BZD and interactive PPI networks topological analysis were used to construct a Venn diagram. KEGG was then performed to further analyze the genes located at the intersections of the BZD target genes and the PPI networks’ topological analyses. Finally, the key pathways associated with BZD activity against OA were identified.

### Experiment 4. Verification of the Involvement of Targeted Pathways Using Western Blot Analysis

Based on the NPA results, western blot analysis was used to verify the targeted pathway’s involvement, which was determined to be NF-κB signaling (see the *Results* and *Discussion* section). Pyrrolidine dithiocarbamate (PDTC), an inhibitor of the NF-κB signaling pathway, was selected as a positive control. Chondrocytes were divided into a control group, a model group (LPS 10 ng/ml), a BZD group (BZD 50 µg/ml + LPS 10 ng/ml), and a PDTC group (PDTC10 µM + LPS 10 ng/ml), and treated for 8 h. The western blot procedure was done as described above. The primary antibodies used were NF-κB p65 (ab16502, Abcam, United States), IKK-α (ab32041, Abcam, United States), IKK-β (ab124957, Abcam, United States), MMP-3 (ab52915, Abcam, United States), Collagen II (ab34712, Abcam, United States), and GAPDH (5174s, Cell Signaling Technology, United States).

### Statistical Analysis

SPSS 20.0 software (SPSS Inc., Chicago, IL, United States) was used for statistical analyses. The results are presented as the mean ± standard deviation (SD). Data were analyzed by a two-way analysis of variance followed by Bonferroni’s posthoc correction for multiple comparisons. All the experiments were repeated independently three times. *p* < 0.05 was considered to be significantly different.

## Results

### Experiment 1. Bushen Zhuangjin Decoction Quality Control

Three main components were identified in the BZD extract using HPLC: monoglucoside (peak 1), paeoniflorin (peak 2), and asperosaponin VI (peak 3) (Supplementary Figure S1). Thus, the concentrations of monoglucoside, paeoniflorin, and asperosaponin VI were used as quality control markers.

### Experiment 2. Analysis of the Therapeutic Effects of Bushen Zhuangjin Decoction

In this experiment, changes of MMP-9, IL-6 and NF-κB p65 levels were used as preliminary indicators of the activation (suppression) of the NF-κB signaling pathway.

#### Bushen Zhuangjin Decoction Decreases MMP-9 and IL-6 Levels in LPS-Treated Chondrocytes

**FIGURE 1:**
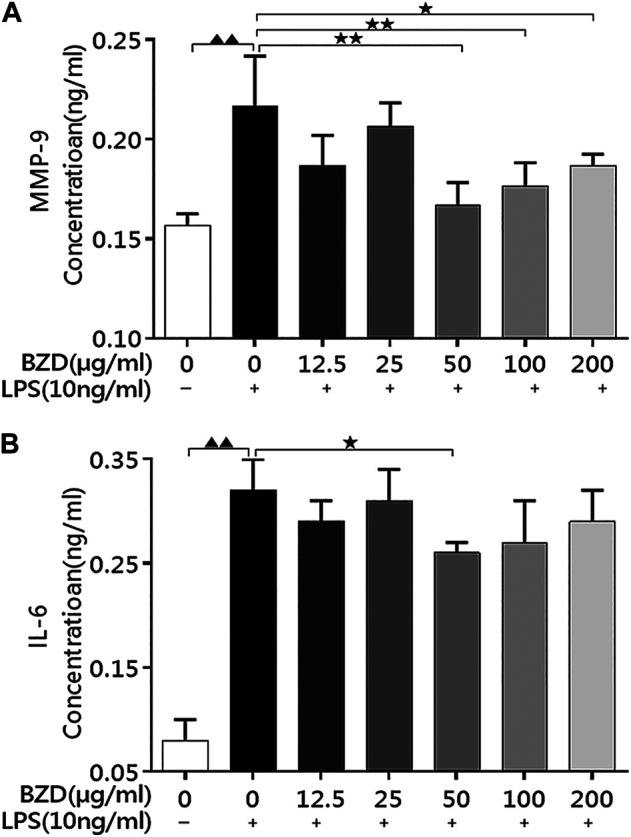
BZD administration decreased MMP-9 and IL-6 levels in LPS-exposed chondrocytes. **(A)** The MMP-9 levels in the culture medium after treatment with different concentrations of BZD for 8 h. **(B)** The IL-6 levels in the culture medium after treatment with different concentrations of BZD for 8 h ^▲▲^ means *p* < 0.01, vs. intact control; * means *p* < 0.05 and ** means *p* < 0.01, vs. LPS-exposed chondrocytes.

LPS exposure significantly upregulated MMP-9 (Figure 1A) and IL-6 (Figure 1B) levels. When BZD was administrated for 8 h, the expression of both proteins decreased, whereas treatment with 50 µg/ml resulted in a significant reduction (*p* < 0.01 for MMP-9 and *p* < 0.05 for IL-6 vs. LPS-exposed chondrocytes, Figure 1). Therefore, we selected an 8 h exposure of 50 µg/ml BZD for subsequent experiments.

#### Bushen Zhuangjin Decoction Suppresses the Expression of Inflammatory Genes

**FIGURE 2:**
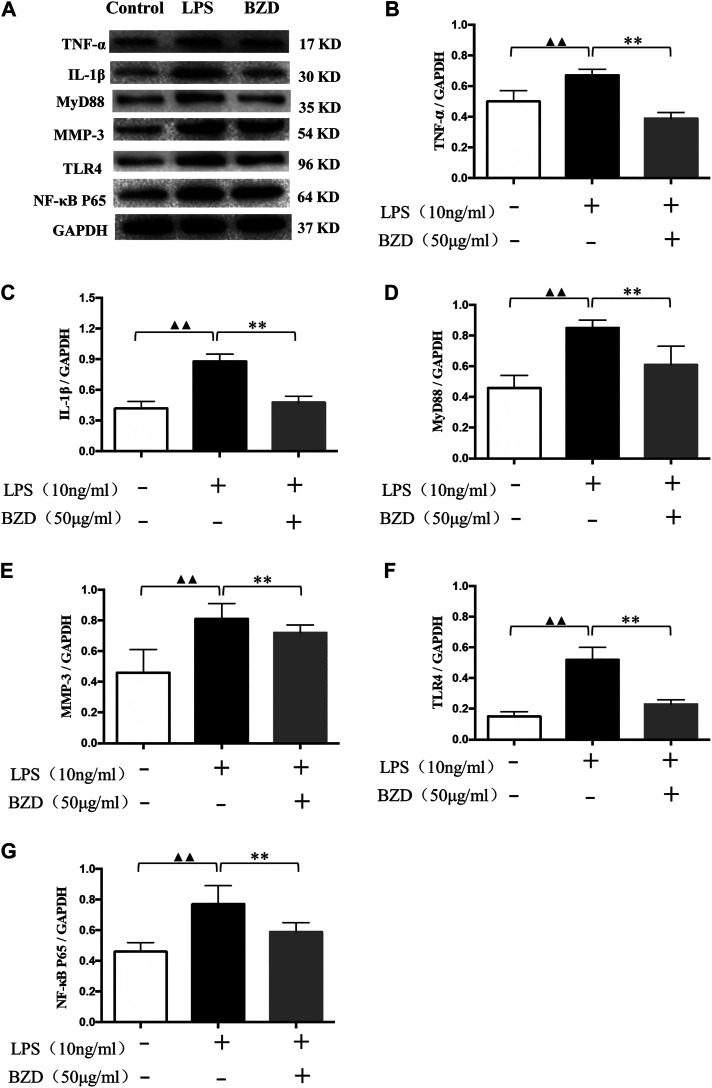
The effects of LPS and BZD exposure on protein expression. **(A)** Representative images of western blot analysis. **(B–G)** Relative protein expression of inflammatory-related genes. TNF-α **(B)**, IL-1β **(C)**, MyD88 **(D)**, MMP-3 **(E)**, TLR4 **(F)**, and NF-κB p65 **(G)** exhibited a similar pattern. Protein expression was significantly upregulated by LPS exposure and significantly downregulated by BZD treatment. ^▲▲^ means *p* < 0.01, vs. intact control; ** means *p* < 0.01, vs. LPS-exposed chondrocytes.

The results of western blot analysis revealed that a number of inflammatory-related genes with different mechanisms, including TNF-α, IL-1β, Myd88, MMP-3, TLR4, and NF-κB p65, exhibited similar changes. LPS exposure significantly upregulated protein expression, whereas BZD treatment significantly downregulated expression (Figure 2). The proteins involved in different mechanisms exhibited the same pattern, indicating that the mechanisms of BZD are complicated and multidimensional.

Here, expression of MMP-9, IL-6 and NF-κB p65 significantly upregulated by the LPS exposure, whereas significantly downregulated by BZD treatment, strongly implied that activation (suppression) of the NF-κB signaling pathway plays a role in the effects of LPS exposure (BZD treatment). Therefore, we used NPA to explore relevant targets and pathways.

### Experiment 3. Screening of Bioactive Components and Molecular Targets of BZD Using NPA

#### Compound–Target Network Analysis

**FIGURE 3:**
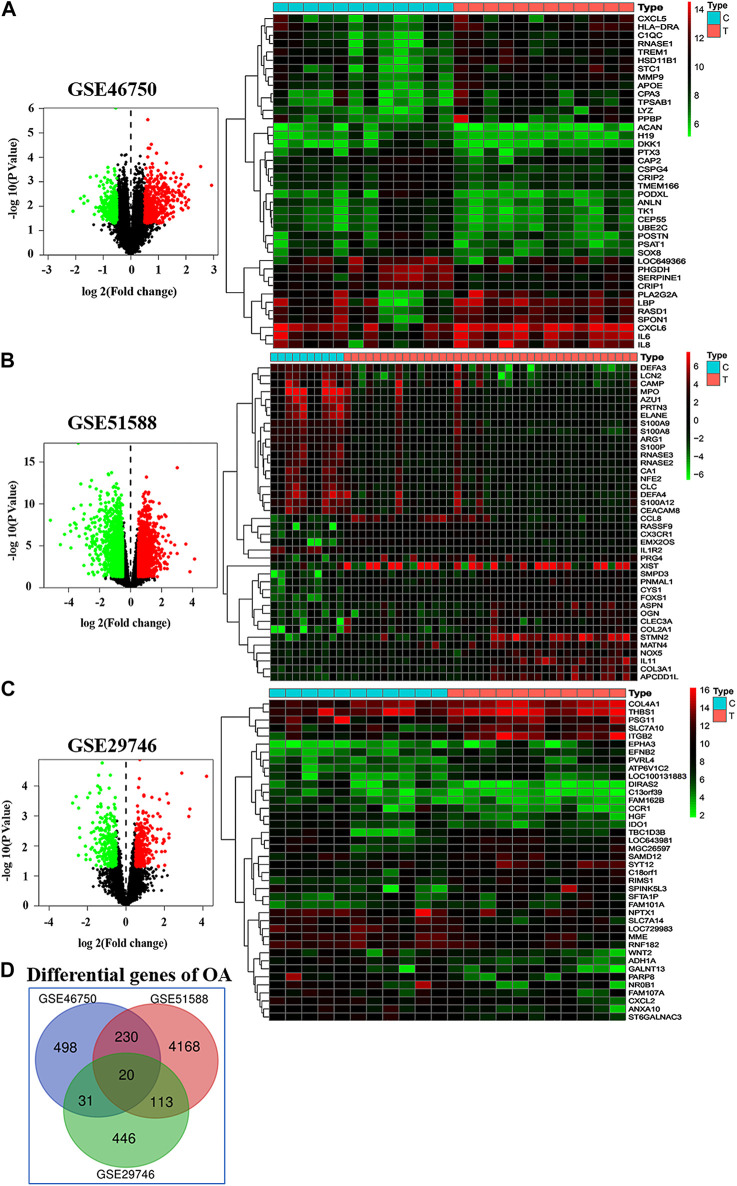
Differentially gene expression in GSE46750, GSE51588, and GSE29746. **(A–C)** In the volcano plot (left column), the abscissa represents the fold changes in gene expression, and the ordinate represents the statistical significance of the variations in gene expression. For GSE46750, there are 469 red dots representing significantly upregulated genes, and 310 green dots representing significantly downregulated genes **(A)**. For GSE51588, there are 2,230 red dots and 2,301 green dots **(B)**. For GSE29746, there are 291 red dots and 319 green dots **(C)**. In the heat map (right column), the red pixels represent significantly upregulated genes, and the green represents significantly downregulated genes. **(D)** A total 20 OA associated targets are located at the intersection of GSE46750, GSE51588, and GSE29746 These include CCNA2, CKS2, CCNF, KIF11, ETS2, FRMD6, CENPN, POLQ, CCR1, CAP2, MATN2, TPX2, CENPE, PDE4B, DLGAP5, CD33, TNFSF10, ANGPTL2, LTC4S, and PMEPA1.

A total of 779, 4,531, and 610 OA-related targets were identified from the GEO database for GSE46750, GSE51588, and GSE29746. The volcano plot and heat maps are shown in Figures 3A–C. A total of 20 OA-related targets were located at the intersection of GSE46750, GSE51588, and GSE29746 (Figure 3D). After the duplications were removed, the remaining 98 compounds were considered to be candidate compounds from the TCM systems pharmacology database (Table 1).

**TABLE 1:** The candidate compounds for BZD as selected by NPA.

ID	Name	OB	DL	Medical plants
MOL000359	Sitosterol	36.91	0.75	RRP, RD, FC, RPA
MOL000449	Stigmasterol	43.83	0.76	RRP, RAS, RAB, FC
MOL000358	Beta-sitosterol	36.91	0.75	RAS, RD, RAB, FC, CE, RPA
MOL003152	Gentisin	64.06	0.21	RD
MOL009312	(E,E)-3,5-Di-O-caffeoylquinic acid	48.14	0.68	RD
MOL009317	Cauloside A_qt	43.32	0.81	RD
MOL008188	Japonine	44.11	0.25	RD
MOL009322	Sylvestroside III	48.02	0.53	RD
MOL009323	Sylvestroside III_qt	56.47	0.43	RD
MOL001006	Poriferasta-7,22E-dien-3beta-ol	42.98	0.76	RAB
MOL012461	28-norolean-17-en-3-ol	35.93	0.78	RAB
MOL012505	Bidentatoside, ii_qt	31.76	0.59	RAB
MOL012537	Spinoside A	41.75	0.4	RAB
MOL012542	β-ecdysterone	44.23	0.82	RAB
MOL001454	Berberine	36.86	0.78	RAB
MOL001458	Coptisine	30.67	0.86	RAB
MOL000173	Wogonin	30.68	0.23	RAB
MOL002643	Delta 7-stigmastenol	37.42	0.75	RAB
MOL002714	Baicalein	33.52	0.21	RAB
MOL002776	Baicalin	40.12	0.75	RAB
MOL002897	Epiberberine	43.09	0.78	RAB
MOL003847	Inophyllum E	38.81	0.85	RAB
MOL004355	Spinasterol	42.98	0.76	RAB
MOL000785	Palmatine	64.6	0.65	RAB
MOL000085	Beta-daucosterol_qt	36.91	0.75	RAB
MOL000422	Kaempferol	41.88	0.24	RAB, CE, RPA
MOL000098	Quercetin	46.43	0.28	RAB, CE
MOL000273	(2R)-2-[(3S,5R,10S,13R,14R,16R,17R)-3,16-dihydroxy-4,4,10,13,14-pentamethyl-2,3,5,6,12,15,16,17-octahydro-1H-cyclopenta[a] phenanthren-17-yl]-6-methylhept-5-enoic acid	30.93	0.81	P
MOL000275	Trametenolic acid	38.71	0.8	P
MOL000276	7,9(11)-dehydropachymic acid	35.11	0.81	P
MOL000279	Cerevisterol	37.96	0.77	P
MOL000280	(2R)-2-[(3S,5R,10S,13R,14R,16R,17R)-3,16-dihydroxy-4,4,10,13,14-pentamethyl-2,3,5,6,12,15,16,17-octahydro-1H-cyclopenta[a] phenanthren-17-yl]-5-isopropyl-hex-5-enoic acid	31.07	0.82	P
MOL000282	Ergosta-7,22E-dien-3beta-ol	43.51	0.72	P
MOL000283	Ergosterol peroxide	40.36	0.81	P
MOL000285	(2R)-2-[(5R,10S,13R,14R,16R,17R)-16-hydroxy-3-keto-4,4,10,13,14-pentamethyl-1,2,5,6,12,15,16,17-octahydrocyclopenta[a]phenanthren-17-yl]-5-isopropyl-hex-5-enoic acid	38.26	0.82	P
MOL000287	3beta-hydroxy-24-methylene-8-lanostene-21-oic acid	38.7	0.81	P
MOL000289	Pachymic acid	33.63	0.81	P
MOL000290	Poricoic acid A	30.61	0.76	P
MOL000291	Poricoic acid B	30.52	0.75	P
MOL000292	Poricoic acid C	38.15	0.75	P
MOL000296	Hederagenin	36.91	0.75	P
MOL000300	Dehydroeburicoic acid	44.17	0.83	P
MOL001798	Neohesperidin_qt	71.17	0.27	PCRV
MOL001803	Sinensetin	50.56	0.45	PCRV
MOL004328	Naringenin	59.29	0.21	PCRV
MOL005100	5,7-dihydroxy-2-(3-hydroxy-4 methoxyphenyl) chroman-4-one	47.74	0.27	PCRV
MOL005828	Nobiletin	61.67	0.52	PCRV
MOL001494	Mandenol	42	0.19	FC
MOL001495	Ethyl linolenate	46.1	0.2	FC
MOL001771	Poriferast-5-en-3beta-ol	36.91	0.75	FC
MOL002879	Diop	43.59	0.39	FC
MOL002883	Ethyl oleate (NF)	32.4	0.19	FC
MOL003137	Leucanthoside	32.12	0.78	FC
MOL005360	Malkangunin	57.71	0.63	FC
MOL005481	2,6,10,14,18-pentamethylicosa-2,6,10,14,18-pentaene	33.4	0.24	FC
MOL005486	3,4-Dehydrolycopen-16-al	46.64	0.49	FC
MOL005489	3,6-Digalloylglucose	31.42	0.66	FC
MOL005503	Cornudentanone	39.66	0.33	FC
MOL005530	Hydroxygenkwanin	36.47	0.27	FC
MOL005531	Telocinobufagin	69.99	0.79	FC
MOL008457	Tetrahydroalstonine	32.42	0.81	FC
MOL000554	Gallic acid-3-O-(6′-O-galloyl)-glucoside	30.25	0.67	FC
MOL005552	Gemin D	68.83	0.56	FC
MOL005557	Lanosta-8,24-dien-3-ol,3-acetate	44.3	0.82	FC
MOL002058	40957-99-1	57.2	0.62	CE
MOL004367	Olivil	62.23	0.41	CE
MOL000443	Erythraline	49.18	0.55	CE
MOL005922	Acanthoside B	43.35	0.77	CE
MOL006709	AIDS214634	92.43	0.55	CE
MOL007059	3-beta-Hydroxymethyllenetanshiquinone	32.16	0.41	CE
MOL000073	Ent-Epicatechin	48.96	0.24	CE
MOL007563	Yangambin	57.53	0.81	CE
MOL009007	Eucommin A	30.51	0.85	CE
MOL009009	(+)-medioresinol	87.19	0.62	CE
MOL009015	(−)-Tabernemontanine	58.67	0.61	CE
MOL009027	Cyclopamine	55.42	0.82	CE
MOL009029	Dehydrodiconiferyl alcohol 4,gamma′-di-O-beta-D-glucopyanoside_qt	51.44	0.4	CE
MOL009030	Dehydrodieugenol	30.1	0.24	CE
MOL009031	Cinchonan-9-al, 6′-methoxy-, (9R)-	68.22	0.4	CE
MOL009038	GBGB	45.58	0.83	CE
MOL009042	Helenalin	77.01	0.19	CE
MOL009047	(+)-Eudesmin	33.29	0.62	CE
MOL009053	4-[(2S,3R)-5-[(E)-3-hydroxyprop-1-enyl]-7-methoxy-3-methylol-2,3-dihydrobenzofuran-2-yl]-2-methoxy-phenol	50.76	0.39	CE
MOL009055	Hirsutin_qt	49.81	0.37	CE
MOL009057	Liriodendrin_qt	53.14	0.8	CE
MOL002773	Beta-carotene	37.18	0.58	CE
MOL008240	(E)-3-[4-[(1R,2R)-2-hydroxy-2-(4-hydroxy-3-methoxy-phenyl)-1-methylol-ethoxy]-3-methoxy-phenyl]acrolein	56.32	0.36	CE
MOL011604	Syringetin	36.82	0.37	CE
MOL000211	Mairin	55.38	0.78	CE, RPA
MOL001910	11alpha,12alpha-epoxy-3beta-23-dihydroxy-30-norolean-20-en-28,12beta-olide	64.77	0.38	RPA
MOL001918	Paeoniflorgenone	87.59	0.37	RPA
MOL001919	(3S,5R,8R,9R,10S,14S)-3,17-dihydroxy-4,4,8,10,14-pentamethyl-2,3,5,6,7,9-hexahydro-1H cyclopenta [a] phenanthrene-15,16-dione	43.56	0.53	RPA
MOL001921	Lactiflorin	49.12	0.8	RPA
MOL001924	Paeoniflorin	53.87	0.79	RPA
MOL001925	Paeoniflorin_qt	68.18	0.4	RPA
MOL001928	Albiflorin_qt	66.64	0.33	RPA
MOL001930	Benzoyl paeoniflorin	31.27	0.75	RPA
MOL000492	(+)-catechin	54.83	0.24	RPA

OB, oral bioavailability; DL, drug-likeness; RRP, Radix Rehmanniae Preparata; RAS, Radix Angelicae Sinensis; RD, Radix Dipsaci; RAB, Radix Achyranthis Bidentatae; P, Poria; PCRV, Pericarpium Citri Reticulatae Viride; FC, Fructus Corni; CE, Cortex Eucommiae; RPA, Radix Paeoniae Alba.

**FIGURE 4:**
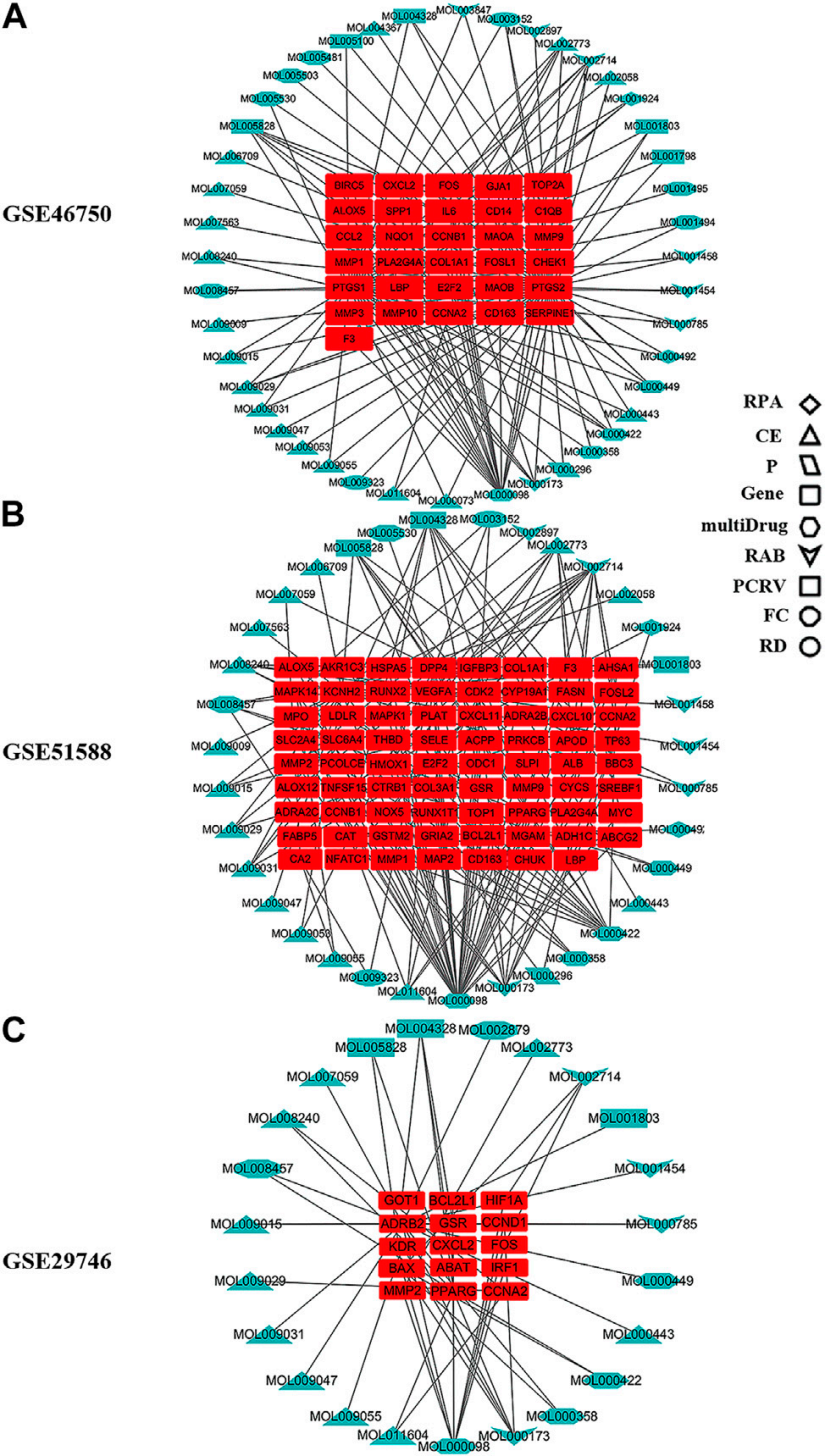
Compound–target network for BZD. The round red rectangles represent targets; the diamond, triangle, parallelogram, hexagon, quadrilateral arrow, rectangle, octagon, and ellipse represent the compounds from RPA, CE, P, multidrug, RAB, PCRV, FC, and RD, respectively. **(A)** GSE46750, **(B)** GSE51588, **(C)** GSE29746.

The compound–target network for BZD was constructed using the screened compounds and their targets (Figure 4). For GSE46750, the network contained 75 nodes (31 compounds for BZD and 44 compound targets) and 119 edges representing compound–target interactions (Figure 4A). For GSE51588, the network included 106 nodes (35 compounds for BZD and 71 compound targets) and 156 edges (Figure 4B). For GSE29746, the network included 38 nodes (23 compounds in BZD and 15 compound targets) and 47 edges (Figure 4C). The largest degrees for the three series were quercetin, which yielded a value of 46.43%.

#### Identification of Candidate Targets for Bushen Zhuangjin Decoction Anti-Osteoarthritis

**FIGURE 5:**
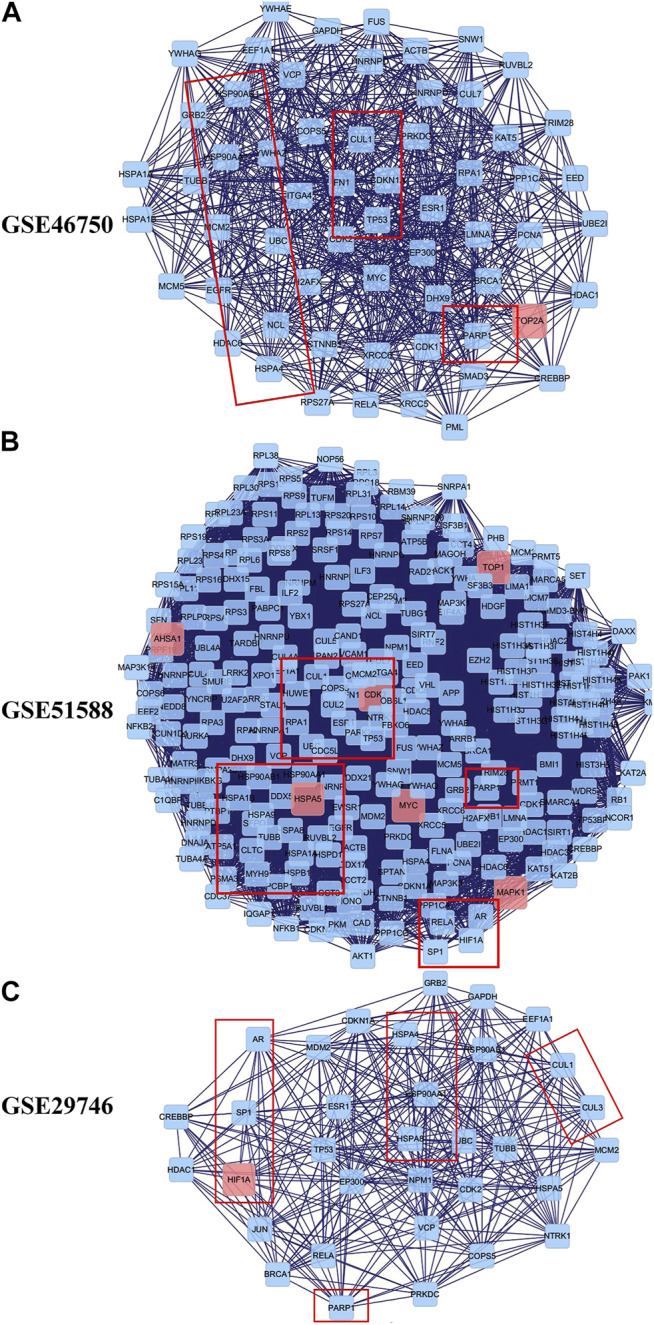
Identification of candidate targets for BZD activity against OA. The interactive PPI network topology analysis of BZD putative targets and OA-related targets were constructed for GSE46750 **(A)**, GSE51588 **(B)**, and GSE29746 **(C)**.

To explore the underlying mechanisms of BZD activity in OA, we merged the PPI networks of the putative BZD targets and OA-related targets to identify a set of candidate targets. For GSE46750, the network consisted of 1,054 nodes and 14,977 edges. The median degree of all nodes was 17, and nodes with more than 61° were identified as significant targets according to a previous study (Zeng et al., 2019). A secondary network consisting of significant (DC > 61) targets for BZD anti-OA activity was then constructed, which contained 139 nodes and 2,932 edges. The median values for BC and CCT were 75.38397 and 0.589744, respectively. The candidate targets were further screened, and 60 targets with BC > 75.38397 and CCT > 0.589744 were identified to generate the final network (Figure 5A). Thus, 60 target genes were eventually identified for BZD anti-OA activity in GSE46750. Similarly, for GSE51588, the network consisted of 3,265 nodes and 80,834 edges. Likewise, the median degree of all nodes was 30, and the nodes with more than 61° were identified as significant targets. The secondary network was constructed by the selection of targets with DC > 61 and contained 837 nodes and 35,888 edges. The median values for BC, CCT, EC, LAC, and NC were 338.4249, 0.517647, 0.023029, 17.30864, and 19.03273, respectively. The candidate targets were further screened and 262 targets with BC > 338.4249, CCT > 0.517647, EC > 0.023029, LAC > 17.30864 and NC > 19.03273 were identified to generate the final network (Figure 5B). Two hundred and sixty-two target genes were eventually identified in GSE46750. For GSE29746, the network consisted of 1,237 nodes and 19,308 edges. The median degree of all nodes was 19, and nodes with more than 61° were considered significant targets. The secondary network with DC > 61 contained 165 nodes and 3,693 edges. The median values for BC, CCT, EC, and LAC were 89.22116, 0.577465, 0.074042, and 15.8, respectively. The candidate targets were further screened and 33 targets with BC > 89.22116, CCT > 0.577465, EC > 0.074042, and LAC > 15.8 were identified to generate the final network (Figure 5C). Thirty-three target genes were eventually identified for GSE29746.

#### Kyoto Encyclopedia of Genes and Genomes and Gene Ontology Enrichment Analysis

We analyzed the GO and KEGG pathway using the Cluster Profiler R package to examine the 31 compound targets in GSE46750, 71 compound targets in GSE51588, and 15 compound targets in the GSE29746 group, as presented in Figure 4. For GSE46750, a total of 281 GO terms were significantly enriched (FDR < 0.05), with 252 associated with the BP, 6 with the CC, and 23 with MF categories. The highly enriched GO terms for the BP, CC, and MF included a response to lipopolysaccharide, response to a molecule of bacterial origin, collagen-containing extracellular matrix, condensed chromosome, endopeptidase activity, and metalloendopeptidase activity. For GSE51588, a total of 551 GO terms were significantly enriched (FDR < 0.05), with 501 associated with the BP, 23 with the CC, and 27 with MF categories. The highly enriched GO terms for the BP, CC, and MF included response to oxidative stress, response to nutrient levels, secretory granule lumen, cytoplasmic vesicle lumen, serine-type endopeptidase activity, and serine-type peptidase activity. For GSE29746, a total of 416 GO terms were significantly enriched (FDR < 0.05), with 378 associated with BP, 7 with the CC, and 31 with MF categories. The highly enriched GO terms for the BP, CC, and MF included regulation of membrane potential, response to alcohol, RNA polymerase II transcription factor complex, nuclear transcription factor complex, heat shock protein binding, and coenzyme binding. The top 20 terms are shown in Supplementary Figures S2–S4.

**FIGURE 6:**
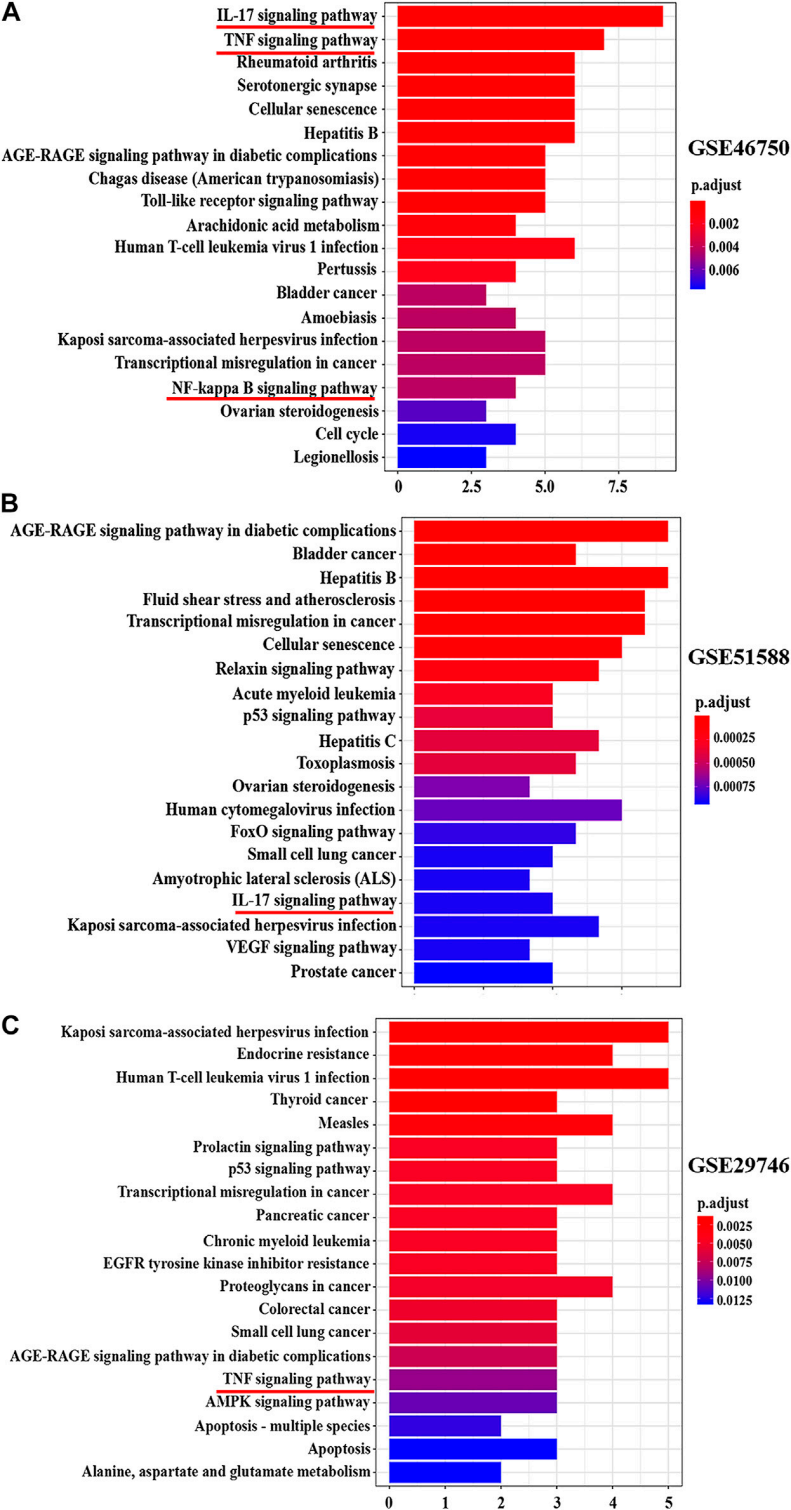
Top 20 KEGG pathway enrichment candidate targets for BZD activity against OA for each expression profile. Pathways with significant changes (FDR < 0.05) were identified. The vertical coordinates represent the KEGG pathway with significant enrichment, and the horizontal coordinates represent the number of differentially expressed genes in each pathway. The color of the bar graph indicates the significance of the enriched KEGG pathway, and the color gradient represents the size of the *p*-value. Inflammatory signaling pathways are underlined with a red bar. **(A)** GSE46750, **(B)** GSE51588, **(C)** GSE29746.

Figure 6 shows the pathways significantly influenced by BZD and OA, as identified by KEGG pathway analysis. For GSE46750, 41 considerably enriched pathways (FDR < 0.05) were identified, including the IL-17 signaling pathway, TNF signaling pathway, rheumatoid arthritis, serotonergic synapse, and cellular senescence. For GSE51588, 75 significantly enriched pathways (FDR < 0.05) were identified, including the AGE-RAGE signaling pathway associated with diabetic complications, hepatitis B, fluid shear stress, and atherosclerosis, transcriptional misregulation in cancer, and cellular senescence. For GSE29746, 44 significantly enriched pathways (FDR < 0.05) were identified, including Kaposi sarcoma-associated herpesvirus infection, human T-cell leukemia virus 1 infection, endocrine resistance, measles, and proteoglycans in cancer. Pathways common to the three series included IL-17, TNF, and NF-κB signaling.

#### Gene-Pathway Network Analysis

**FIGURE 7:**
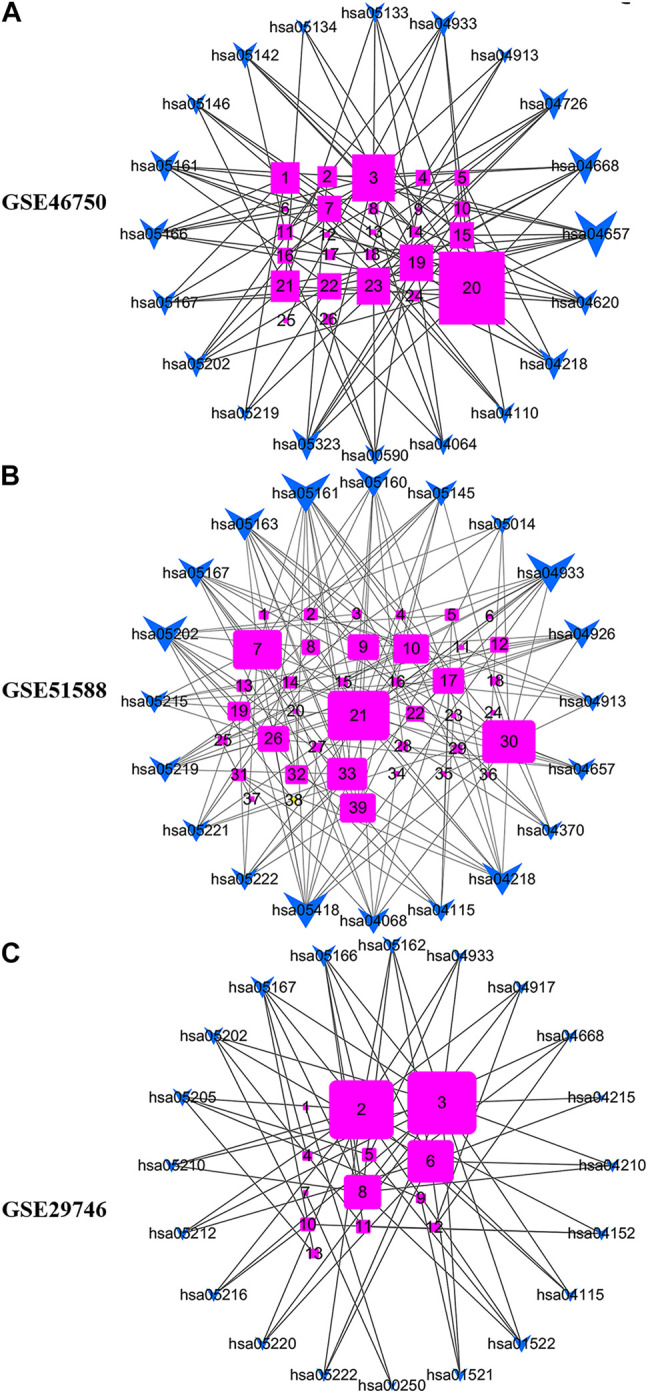
Gene-pathway network for BZD activity against OA. **(A)** For GSE46750, the topological analysis of 20 pathways and 26 genes was performed with degree. 1 = E2F2, 2 = MMP-3, 3 = FOS, 4 = ALOX5, 5 = CHEK1, 6 = BIRC5, 7 = CCNA2, 8 = LBP, 9 = MAOA, 10 = MMP-1, 11 = SERPINE1, 12 = SPP1, 13 = MAOB, 14 = C1QB, 15 = CCL2, 16 = PLA2G4A, 17 = FOSL1, 18 = COL1A1, 19 = CXCL2, 20 = IL6, 21 = CD14, 22 = MMP-9, 23 = PTGS2, 24 = CCNB1, 25 = F3, 26 = PTGS1. **(B)** For GSE51588, the topological analysis of 20 pathways and 39 genes was performed with degree. 1 = CAT, 2 = LDLR, 3 = MPO, 4 = THBD, 5 = IGFBP3, 6 = F3, 7 = CHUK, 8 = CCNA2, 9 = VEGFA, 10 = E2F2, 11 = AKR1C3, 12 = MMP-2, 13 = PLAT, 14 = MMP-1, 15 = COL1A1, 16 = ALOX5, 17 = CDK2, 18 = PLA2G4A, 19 = BCL2L1, 20 = GSTM2, 21 = MAPK1, 22 = PRKCB, 23 = BBC3, 24 = HMOX1, 25 = COL3A1, 26 = MMP-9, 27 = SELE, 28 = CXCL10, 29 = RUNX1T1, 30 = MAPK14, 31 = CCNB1, 32 = NFATC1, 33 = MYC, 34 = SLC2A4, 35 = CYP19A1, 36 = GRIA2, 37 = PPARG, 38 = RUNX2, 39 = CYCS. **(C)** For GSE29746, the topological analysis of 20 pathways and 13 genes was carried out with degree. 1 = GOT1, 2 = CCND1, 3 = BAX, 4 = CXCL2, 5 = MMP-2, 6 = BCL2L1, 7 = ABAT, 8 = FOS, 9 = KDR, 10 = PPARG, 11 = CCNA2, 12 = IRF1, 13 = HIF1A. The fuchsia squares represent target genes, and the blue quadrilateral arrow represents pathways. A large size represents a higher degree.

Figure 7 shows the gene-pathway network constructed based on significantly enriched pathways and genes. For GSE46750, the topological analysis involving 20 pathways and 26 genes was performed based on degree. The network diagram indicated that IL-6 had the highest degree and was, therefore, the core target gene. Several other genes also exhibited a large degree, including FOS, CXCL2, PTGS2, CD14, and E2F2 (Figure 7A). For GSE51588, the topological analysis involving 20 pathways and 39 genes was performed with a degree. The network diagram indicated that MAPK1 had the highest degree and was, therefore, the core target gene. Several other genes also exhibited a large degree, including MAPK14, CHUK, MYC, E2F2, and CYCS (Figure 7B). For GSE29746, the topological analysis involving 20 pathways and 13 genes was performed with a degree. The network diagram indicated that BAX had the highest degree and was, therefore, the core target gene. The other genes that also exhibited a high degree were CCND1, BCL2L1, and FOS (Figure 7C).

**FIGURE 8:**
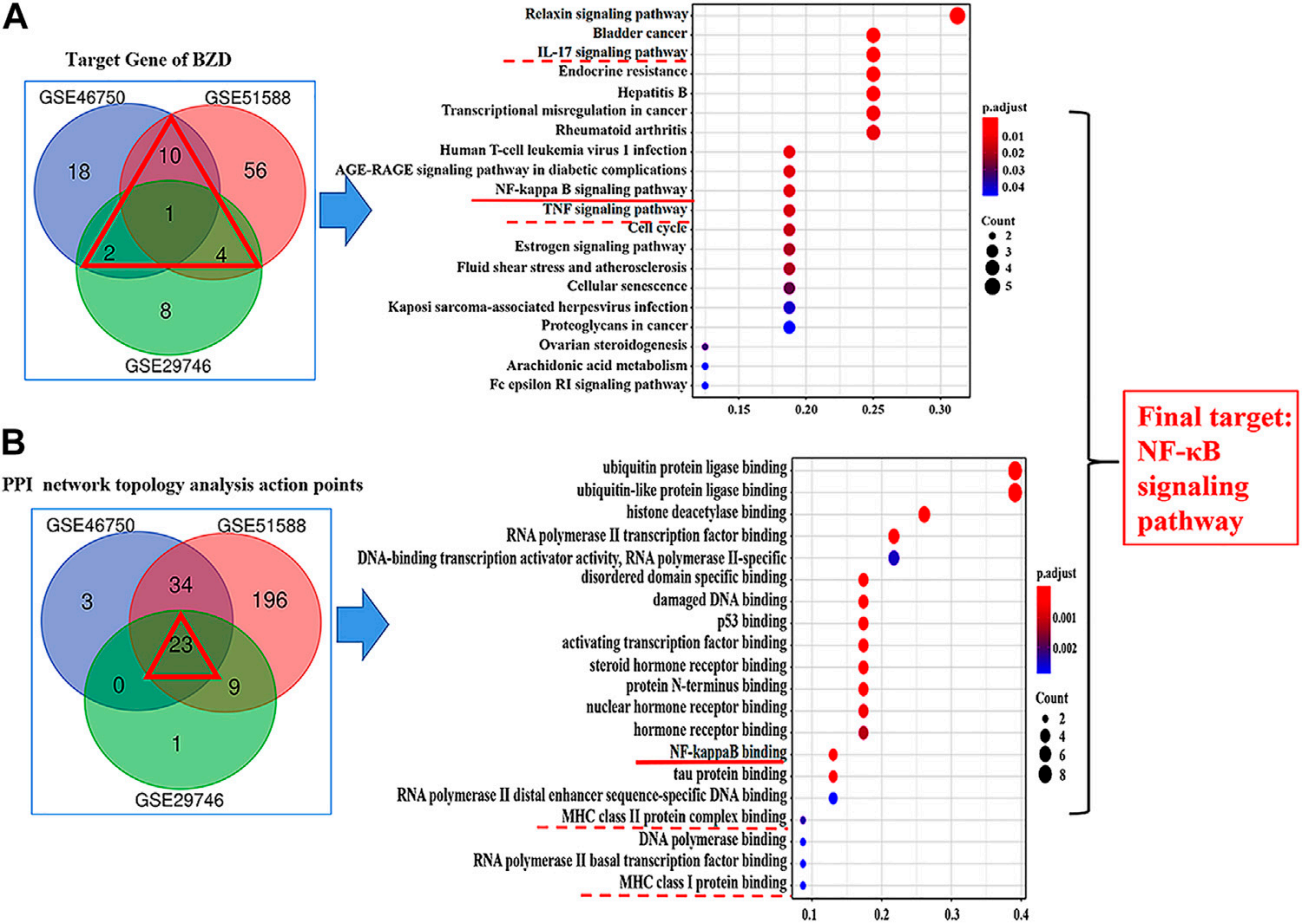
Results of the Gene ontology (GO) and KEGG analysis. **(A)** Results for the target genes of BZD. The intersection is shown by a red triangle, including 17 genes that were submitted to the GO and KEGG analysis. **(B)** Results for the PPI network topology analysis action points. The intersection is shown by a red triangle, including 23 genes that were submitted to the GO and KEGG analysis. The left column shows the results of the Venn diagram analysis. The right column shows the results of the KEGG pathway enrichment of candidate targets based on the Venn diagram analysis. The red under bars represent inflammatory-immune-related pathways. The vertical coordinates represent the KEGG pathway with significant enrichment, and the horizontal coordinates represent the number of differentially expressed genes in each pathway. The color of the bar graph indicates the significance of the enriched KEGG pathway, and the color gradient represents the size of the *p*-value. The size of each dot represents the number of genes.

Figure 8 shows the results of the GO and KEGG analyses based on the Venn diagram analysis. Concerning the target gene analysis results, we found only one gene at the intersection of GSE46750, GSE51588, and GSE29746, namely CCNA2. Therefore, we enlarged the range of analysis to include the genes in the intersections of two profiles (showed as a red triangle). We associated 17 genes with the GO and KEGG analyses (Supplementary Figure S5), whereas three inflammatory-immune-related pathways were identified: IL-17, NF-κB, and TNF signaling pathways (Figure 8A). As for the results of the PPI network topology analysis action points, a total of 23 genes were located at the intersection of the three series and were subjected to GO and KEGG analysis (Supplementary Figures S6A–D). Three inflammatory-immune-related molecular functions were identified: NF-κB binding, MHC class II protein complex binding and MHC class I protein complex binding (Figure 8B). Twelve pathways converged at the intersection of GSE46750, GSE51588, and GSE29746. KEGG enrichment analysis revealed that they represented IL-17, NF-κB, and TNF signaling pathways (Supplementary Figure S6D). Based on the above results, we determined that the NF-κB signaling pathway would be the key target for further investigation.

### Experiment 4. Verification of the Involvement of the NF-κB Signaling Pathway

**FIGURE 9:**
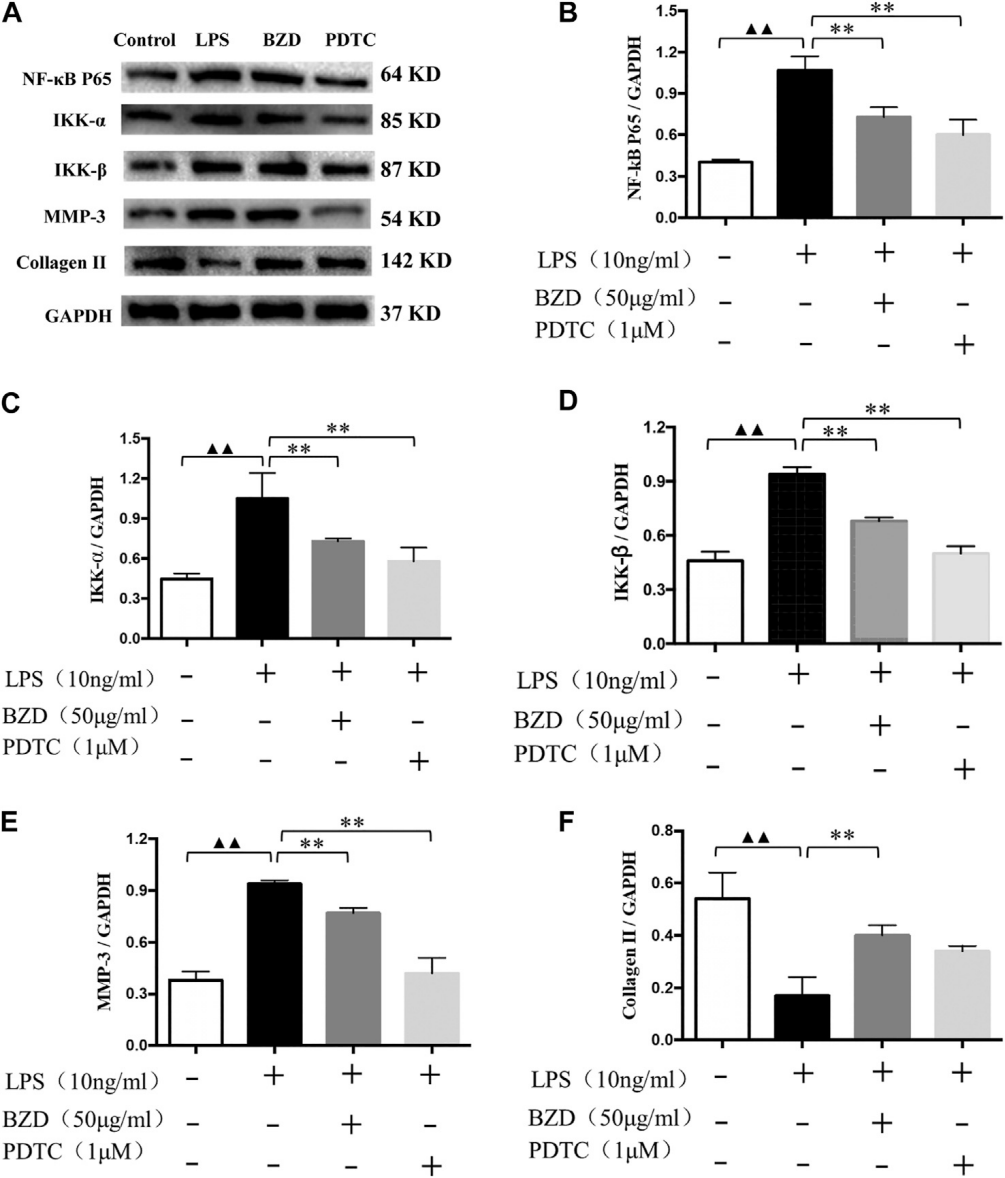
Verification of the targeted NF-κB signaling pathway. **(A)** Representative images of the western blot analysis. **(B–F)** Protein expression affected by LPS, BZD, and PDTC. LPS significantly upregulated the expression of NF-κB p65 **(B)**, IKK-α **(C)**, IKK-β **(D)**, MMP-3 **(E)**. These proteins were significantly downregulated by the administration of BZD. Treatment of the positive control with PDTC downregulated these genes and exhibited significant effects on NF-κB p65, IKK-β, and MMP-3. LPS significantly downregulated the expression of Collagen II **(F)**. It was upregulated considerably by BZD treatment. Treatment with PDTC exhibited the same tendency but did not reach a significant difference. ^▲▲^ means *p* < 0.01, vs. intact control; ** means *p* < 0.01, vs. LPS-exposed chondrocytes.

The western blot analysis results revealed the expression of biomarkers affected by LPS, BZD, and PDTC, an inhibitor of the NF-κB signaling pathway. NF-κB P65, IKK-α, IKK-β, and MMP-3 exhibited similar expression patterns. They were upregulated by LPS exposure and downregulated by treatment with BZD and PDTC. The BZD group exhibited significant differences in all genes, whereas PDTC exhibited significant differences in NF-κB p65, IKK-β, and MMP-3. By contrast, Collagen II was downregulated by LPS exposure but upregulated by BZD and PDTC. Only the BZD group exhibited a significant difference. Therefore, the involvement of the NF-κB signaling pathway was confirmed (Figure 9).

## Discussion

In this study, we tested our hypothesis, namely NF-κB signaling pathway plays a key role in the mechanisms of BZD for treating OA by comparing the NPA results with conventional methods. In the first experiment, we identified the components of BZD using HPLC. In the second experiment, we performed a preliminary observation regarding the therapeutic effects of BZD in an OA cellular model created by LPS exposure. We identified several inflammatory-related genes that are activated by LPS exposure and suppressed by BZD. These genes are associated with different mechanisms; thus, the preliminary observation indicated that the complicated and multifold mechanisms involved in the effects of BZD on OA might be associated with multi-components, multi-targets, and multi-pathways. In the third experiment, we performed a standard NPA for BZD and identified 98 candidate compounds that were associated with OA. KEGG and GO enrichment analyses identified three inflammatory signaling pathways, IL-17, TNF, and NF-κB that were associated with BZD activity. We then performed a Venn diagram analysis consisting of the target genes and the PPI network topology analysis action point. The results indicated that the NF-κB signaling pathway plays a key role in the effects of BZD. These results were verified in the fourth experiment by indirectly verifying NF-κB signaling activity by western blot analysis. We found that BZD indeed contributed to reduced expression of NF-κB related genes, which were, conversely, upregulated by LPS exposure. Thus, the role of the NF-κB signaling pathway was confirmed. To our knowledge, this is the first study to utilize conventional experiments in combination with NPA to identify the key role of NF-κB signaling pathway in the trerapeutic effects of BZD against OA. These findings contribute to better understanding the pharmacological mechanisms of BZD for treating OA. Meanwhile, Our results indicate that NPA is a powerful tool to identify molecular targets incomplex herbal formulation commonly used in TCM.

### Experiment 2: Preliminary Indicators of Role of NF-κB Signaling Pathway

It is known that expression of cytokines like MMP-9 and IL-6 is under the control of NF-κB signaling pathway (Epanchintsev et al., 2015). Our previous study using LPS-treated chondrocytes also found that MMP-9 and IL-6 were significantly upregulated by LPS exposure (Li et al., 2020b). We therefore believe that upregulation (downregulation) of MMP-9 and IL-6 can be used as an “indicator” of activation (suppression) of the NF-κB signaling pathway. We therefore first observed the changes of MMP-9 and IL-6. Our preliminary results revealed that MMP-9 and IL-6 levels were significantly increased by LPS exposure and decreased by BZD (Figure 1). MMP-9 and IL-6 are downregulated factors of NF-κB signaling pathway, perpetuating in OA by modulating inflammatory and catabolic mediators. MMP-9 degrades the collagenase fragments of Collagen II (Alves et al., 2014) by activation of the NF-κB signaling pathway. IL-6, as a pro-inflammatory cytokines, is also relevant to activation of the NF-κB signaling pathway (Pistolic et al., 2009; Liu et al., 2015). The changes of MMP-9 and IL-6 were in agreement with our previous studies in rat (Li et al., 2020b) and in rabbit (Wu G. et al., 2019), which contribute to exacerbating progression of OA. In addition, TNF-α, IL-1β, MyD88, MMP-3, TLR4, and NF-κB p65 were significantly upregulated by LPS, but suppressed by BZD treatment (Figure 2). It is known that IL-6, IL-1β, and TNF-α can induce the expression of MMPs, particularly MMP-3, MMP-9, and MMP-13, and inhibits the synthesis of Collagen II. These proteins play pivotal roles in cartilage matrix degeneration in OA. Pro-inflammatory cytokines such as IL-1β and TNF-α may mediate chondrocyte degeneration, which is associated with OA (Kapoor et al., 2011; Fei et al., 2019). MMP-3 directly degrades the extracellular matrix and indirectly affects the degeneration of the extracellular matrix by activating other latent MMPs (Honsawek et al., 2013). TLR4, MyD88, and NF-κB p65 represent another group of inflammatory-related genes that are associated with the TLR4/MyD88/NF-κB signaling pathway. Activation of TLR4 stimulates the MyD88 adaptor protein, resulting in IκB kinase activation and phosphorylation. This causes the release of cytosolic sequestered NF-κB p65 subunits and their translocation to the nucleus. NF-κB p65 activates gene transcription and protein synthesis of various inflammatory factors to regulate the inflammatory response. Changes in the expression of these genes by BZD exposure indicate that its effects and mechanisms of action are complicated and pleiotropic. However, the changes of MMP-9, IL-6, and the related genes stongly implied the key role of the NF-κB signaling pathway. We therefore further investigated the relevant genes and pathways using NPA.

Our analysis of the Gene-pathway network suggests that IL-6, MAPK1, and BAX exhibited the maximum degree, and may represent core targets for the GSE46750, GSE51588 and GSE29746 profiles. The other top three genes for GSE46750 (FOS, CXCL2, and PTGS2), GSE51588 (MAPK14, CHUK, and MYC), and GSE29746 (CCND1, BCL2L1, and FOS) were selected as key target genes (Figure 7). Comparing the results of experiment 2 with NPA, we confirmed increased levels of MMP-3, MMP-9, and IL-6 in LPS-exposed chondrocytes, whereas BZD decreased all levels of the proteins. MMP-3 was excluded from the top genes of GSE46750, and MMP-9 was excluded from GSE46750 and GSE51588. Genes belonging to the MMP family were identified as key targets of BZD in all three profiles. Moreover, further GO analysis revealed that these genes were associated with metalloendopeptidase and metallopeptidase activity, as shown in Supplementary Figure S5C. The other genes should be verified in future studies.

### Identification and Verification of the Key Role of NF-κB Signaling Pathway

From the preliminary results of experiment 2, we supposed the key role of the NF-κB signaling pathway in the effects of BZD against OA, thus we performed the KEGG pathway enrichment analysis. The results identified 41 pathways in GSE46750, 75 pathways in GSE51588, and 44 pathways in the GSE29746 profiles. Inflammatory-related signaling pathways, including IL-17, TNF, and NF-κB signaling pathways, were identified from the intersection of the three profiles (Supplementary Figure S6D). The IL-17, TNF, and NF-κB signaling pathways were included in the top 20 signaling pathways of GSE46750, the IL-17 signaling pathway was included in the top 20 signaling pathways of GSE51588, and the TNF signaling pathway was included in the top 20 signaling pathways of GSE29746 (Figure 6). Thus, the IL-17, TNF, and NF-κB signaling pathways were identified as the top inflammatory-related signaling pathways associated with OA. With respect to the interactions of these pathways, activation of IL-17 triggers the activation of NF-κB (Schwandner et al., 2000). Similarly, activation of NF-κB is mediated by the activation of TNF (Jackson-Bernitsas et al., 2007), which subsequently induces activation of NF-κB (Mukhopadhyay et al., 2002; Jaco et al., 2017). Therefore, we hypothesized that the NF-κB signaling pathway is downstream of the IL-17 and TNF signaling pathways. The results of the Venn diagram analysis between the target gene and PPI network topology analysis action point also found that only the NF-κB signaling pathway was common to both analyses (Figure 8). Based on these results, we confirmed that the NF-κB signaling pathway contributes to the observed effects of LPS, BZD, and PDTC by measuring the expression of three proteins associated with the NF-κB signaling pathway. A final verification was performed using a traditional western blot analysis (Experiment 4). The expression of NF-κB p65, IKK-β, and MMP-3 was attenuated by BZD and PDTC treatment, thus confirming a key role for the NF-κB signaling pathway in the effects of BZD in OA (Figure 9).

### The Methodology of NPA

We first used three OA-related expression profiles for NPA: GSE46750, GSE51588, and GSE29746. The compound–target networks for BZD were constructed using 44, 35, and 23 compounds, and 31, 71, and 15 compound targets for GSE46570, GSE51588, and GSE29746. Quercetin, wogonin, baicalein, and nobiletin acted on 21, 6, 6, and 6 targets in GSE4750, respectively. Quercetin, baicalein, kaempferol, and wogonin acted on 40, 13, 12, and 9 targets in GSE51588, respectively. Lastly, quercetin, wogonin, naringenin, and baicalein acted on 10, 5, 4, and 3 targets in GSE29746, respectively. Therefore, quercetin, wogonin, and baicalein were considered to be crucial pleiotropically active compounds associated with BZD activity. However, these compounds were not detected in BZD by HPLC analysis.

The PPI networks established for the BZD putative targets and OA-related targets were structured and merged to obtain candidate targets for BZD activity in OA. To achieve more accurate targets, 3, 6, and 5 parameters, including DC and BC, were set to screen nodes, and then to structure them into a new network. Finally, 60, 262, and 33 targets were identified from the GSE46750, GSE51588, and GSE29746 profiles, respectively (Figure 5). There were 23 common action points in the three working networks, including CUL, TP, HSP, and RARP types (Figure 8B).

Biological information on putative BZD targets was analyzed. The targets of BZD anti-OA activity included genes associated with the BP, CC, and MF by GO enrichment analysis. Our data also revealed that BZD regulates some BPs. For example, an effect on reducing cartilage degeneration may be associated with the suppression of the inflammatory response (Figure 6; Supplementary Figures S2–S4). A gene-pathway network was then constructed to investigate the core and key target genes for BZD anti-OA activity (Figure 7). Finally, a Venn diagram analysis was performed to identify the intersections shared among the three OA-related profiles.

### Regarding the Compounds: Experiment 1 vs. NPA

Monoglucoside, paeoniflorin, and asperosaponin VI were selected as quality control markers for the BZD extract because they have been verified as quality control markers for FC, RPA, and RD in the pharmacopoeia of the People’s Republic of China (2015 version). Our HPLC results identified monoglucoside, paeoniflorin, and asperosaponin VI (Supplementary Figure S1), confirming these three compounds as quality control markers for the BZD extract. NPA identified 98 candidate compounds for the GSE46750, GSE51588, and GSE29746 profiles (Table 1) that were associated with OA. Interestingly, we found that paeoniflorin was included from the list of these candidate compounds, despite representing a core role in the effects of BZD on OA. The OB and DL values of paeoniflorin were 53.87 and 0.79, respectively.

Monoglucoside and asperosaponin VI were not included in the list of these candidate compounds. This result indicates that conventional methods, such as simply selecting certain compounds listed in the pharmacopoeia as pharmacologic quality control markers, may have certain limitations. Our findings suggest that the following processes may be a more reasonable method to identify the pharmacologic quality of control markers, particularly for complex herbal formulations with many ingredients. First, use NPA to identify the core bioactive compounds. Then, compare these compounds with the classical pharmacopoeia. Finally, identify the pharmacologic quality control markers. These processes require further verification in future studies using more complex herbal formulations.

Overall, the results of NPA, along with the conventional experiments, suggest that the mechanisms of BZD are complex and include multiple compounds and pathways, with NF-κB signaling playing a key role. Suppression of the NF-κB signaling pathway might be a key mechanism related to chondrocyte apoptosis, which is associated with cartilage degeneration.

## Conclusion

In the present study, we used a set of four sequential experiments to identify and verify the key role of the NF-κB signaling pathway in the BZD efficacy against OA. Our results also indicate that NPA is a powerful toolfor exploring the molecular targets of complex herbal formulations and is useful to guide future studies of “target” compounds, genes, and pathways.

## Data Availability

The original contributions presented in the study are included in the article/Supplementary Material, further inquiries can be directed to the corresponding authors.
