# Supplementary material for: Identification of the Key Role of NF-κB Signaling Pathway in the Treatment of Osteoarthritis With Bushen Zhuangjin Decoction, a Verification Based on Network Pharmacology Approach
Source: Front Pharmacol. 2021 Apr 12;12:637273. doi: 10.3389/fphar.2021.637273 (PMC8072665; doi:10.3389/fphar.2021.637273)
Supplement: Supplementary file 2 [file datasheet2.docx]

**Supplementary Materials**


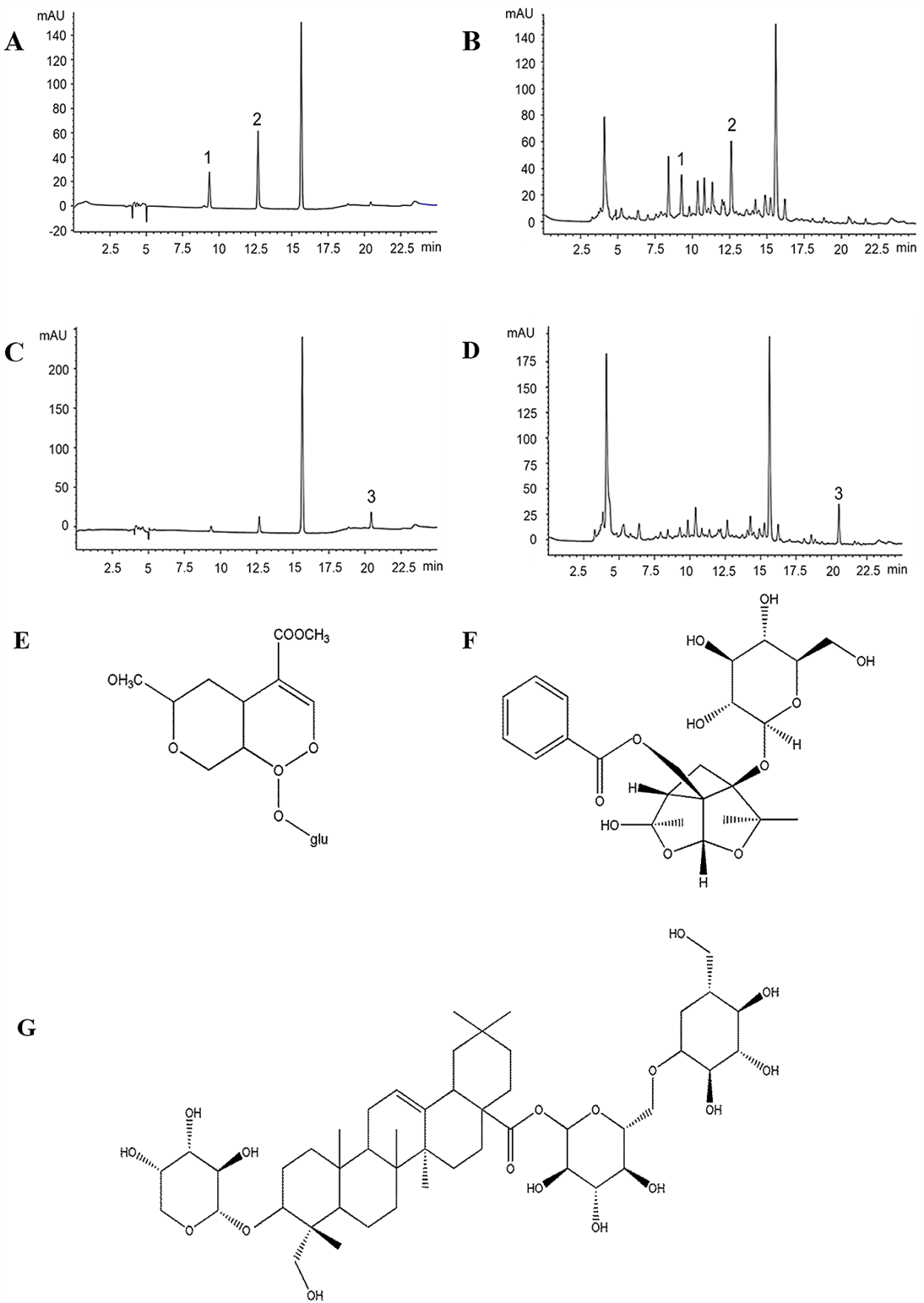


**Figure S1. Main components identified by quality control of BZD extracts using high-performance liquid chromatogram (HPLC) assay.**

A. The liquid chromatogram of the reference substance was composed of 2 peaks at 230 nm. Peak 1: monoglucoside, Peak 2: paeoniflorin.

B. The two peaks were also observed in the liquid chromatogram of the BZD extract at 230 nm.

C. The liquid chromatogram of the reference substance was composed of 1 peak at 212 nm. Peak 3: asperosaponin VI.

D. Peak 1 was also observed in the liquid chromatogram of the BZD extract at 212 nm.

E-G. The structural formula of monoglucoside (E), paeoniflorin (F), and asperosaponin VI (G).


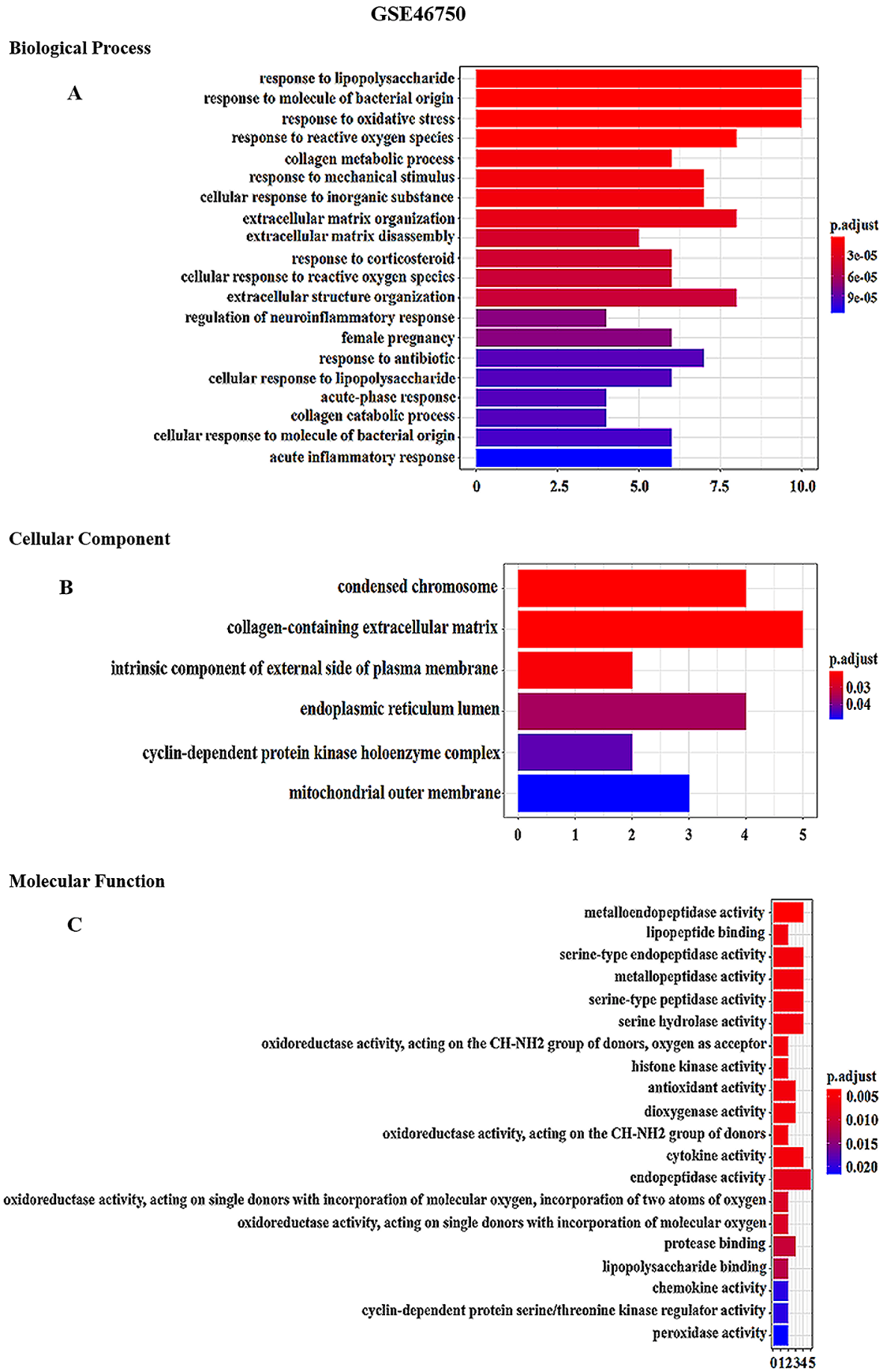


**Figure S2.** **Gene ontology (GO) terms for the candidate targets of BZD activity in OA for GSE46750.**

A. Biological process, B. Cellular component, C. Molecular function.

The top 20 GO functional categories with FDR < 0.05 are shown.


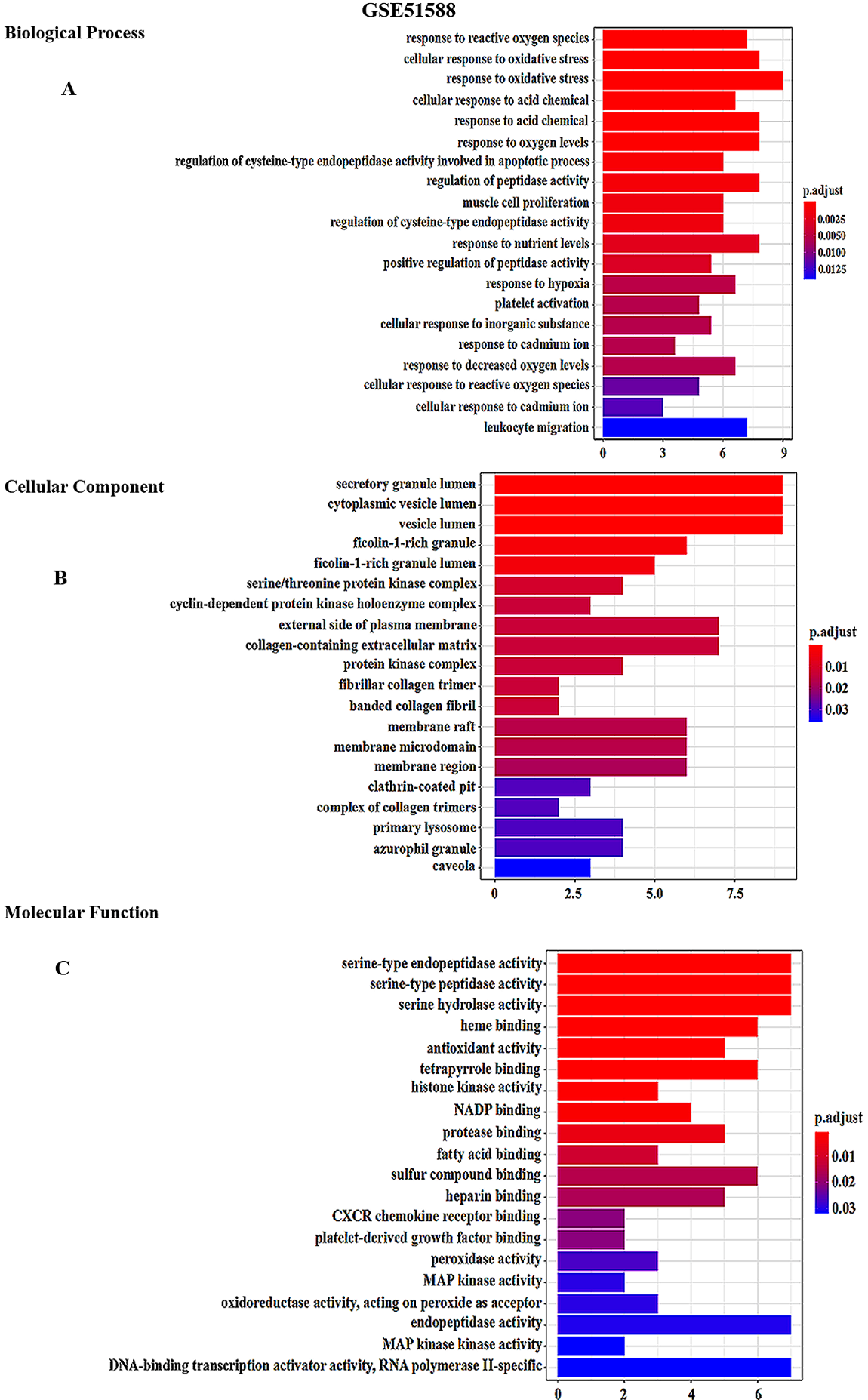


**Figure S3.** **Gene ontology (GO) terms of candidate targets of BZD activity in OA for GSE51588.**

A. Biological process, B. Cellular component, C. Molecular function.

The top 20 GO functional categories with FDR < 0.05 are shown.


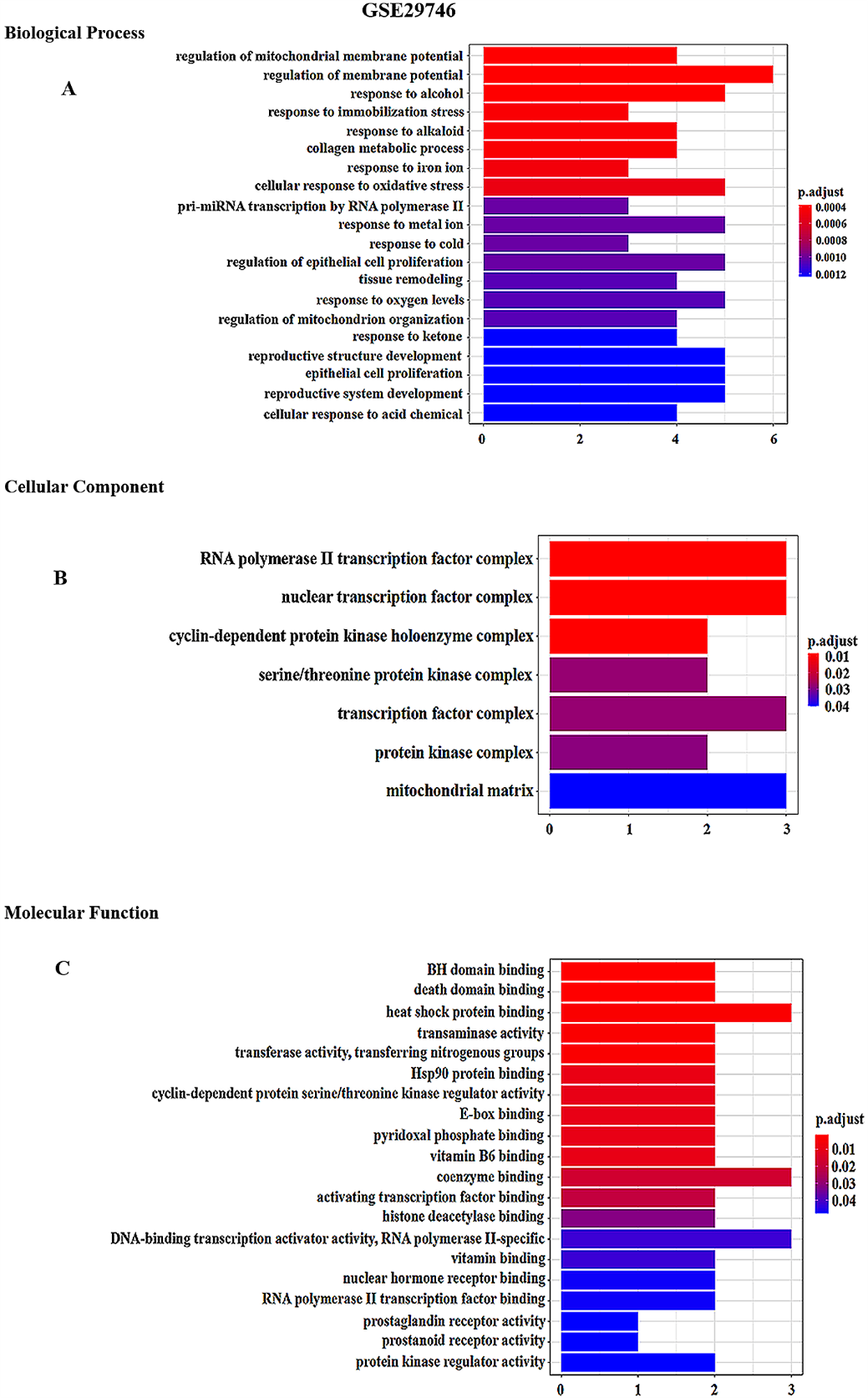


**Figure S4.** **Gene ontology (GO) terms of candidate targets of BZD activity in OA for GSE29764.**

A. Biological process, B. Cellular component, C. Molecular function.

The top 20 GO functional categories with FDR < 0.05 are shown.

**
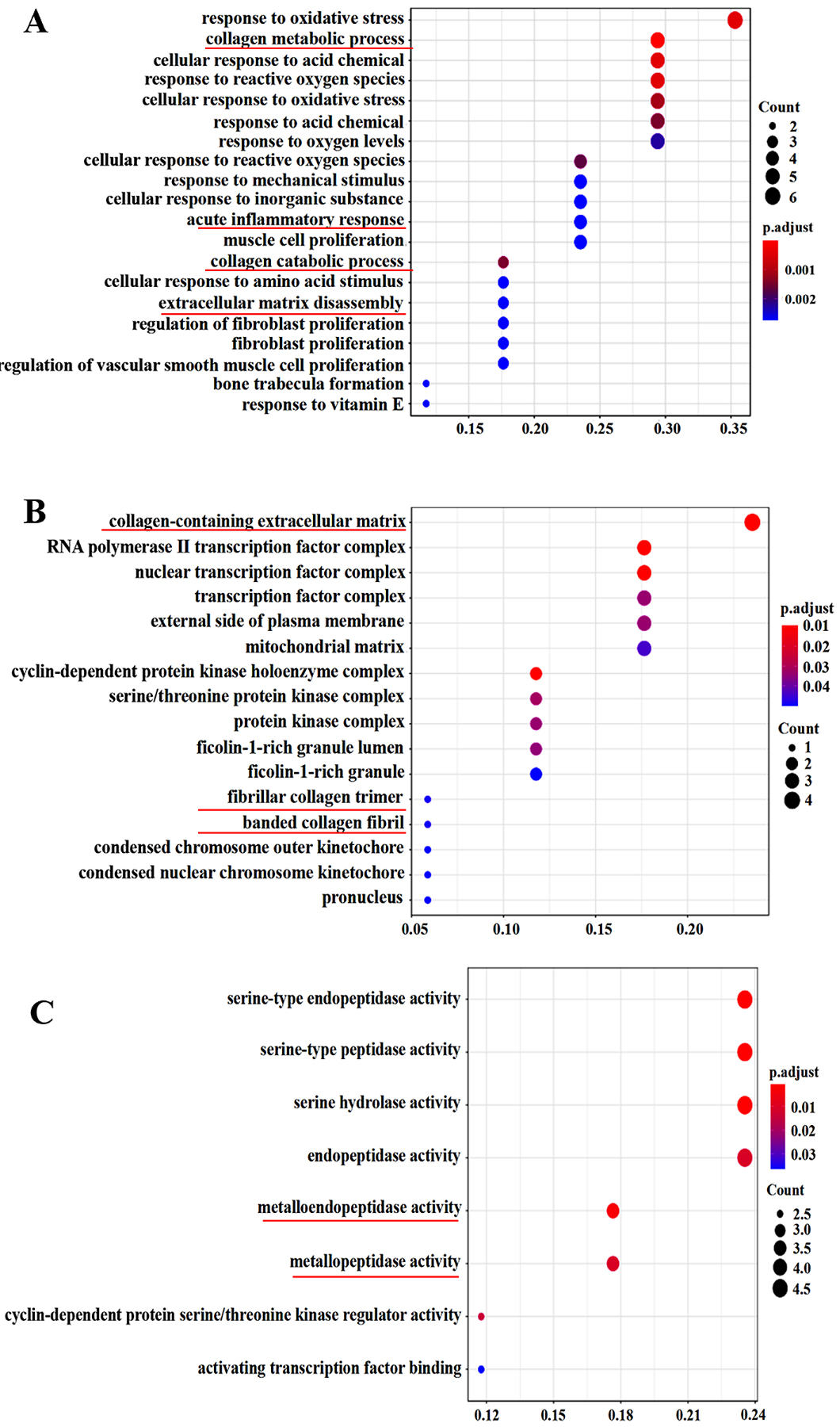
**

**Figure S5. Seventeen genes at the intersection were analyzed by Gene ontology (GO) for BZD activity against OA for GSE46750, GSE51588, and GSE29746.**

A. Biological process, B. Cellular component, and C. Molecular function. Top 20 GO functional categories with FDR < 0.05 are presented.

**
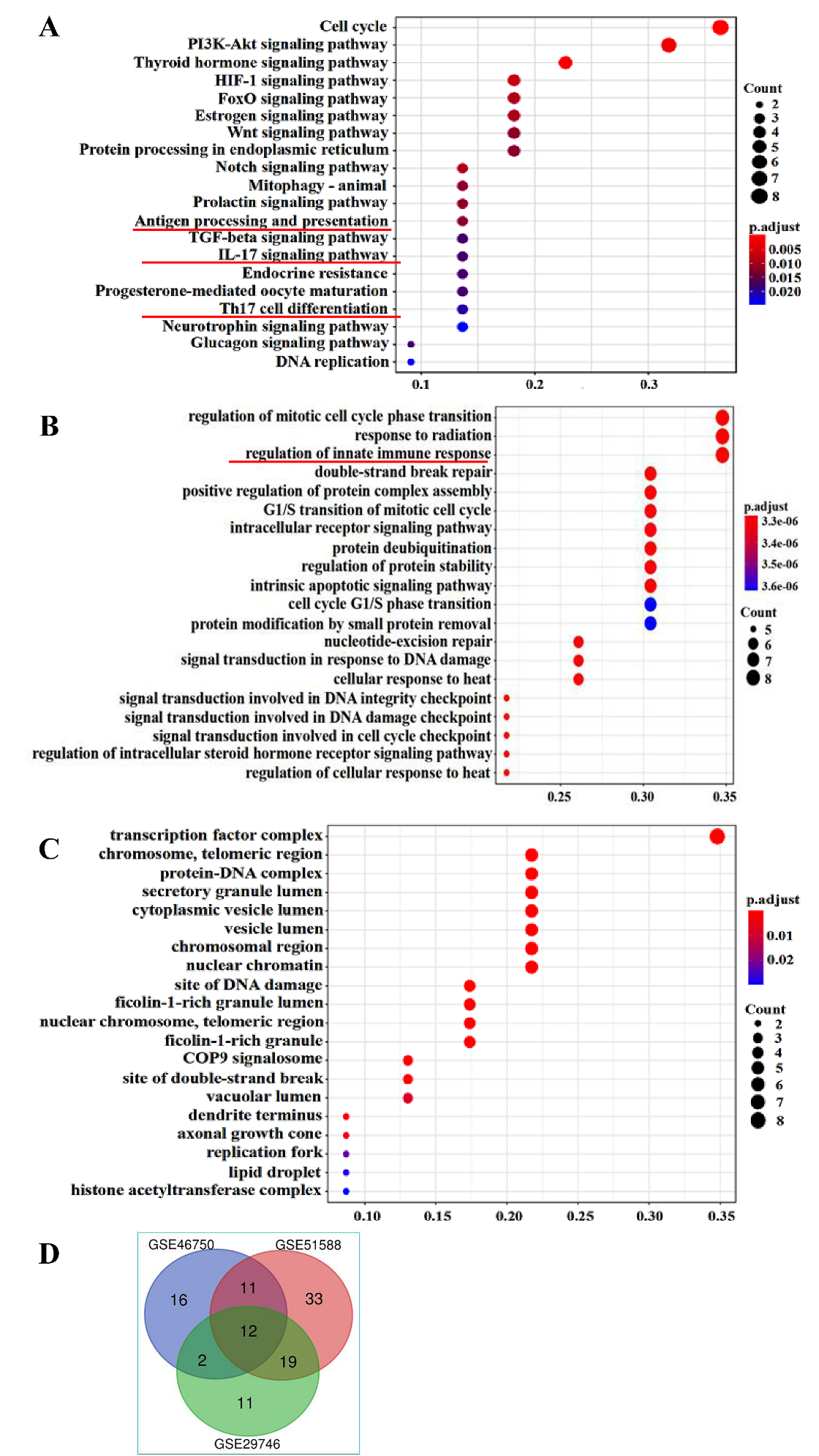
**

**Figure S6. The total 23 genes and 12 pathways at the** **GSE46750, GSE51588, and GSE29746 intersection were analyzed by KEGG.**

A. Action points obtained from the PPI network topology analysis were submitted for the Venn diagram analysis. A total of 23 genes at the intersection from the GSE46750, GSE51588, and GSE29746 profiles were analyzed by KEGG. There were three immune inflammation-related signaling pathways in the top 20 list of pathways, including antigen processing and presentation, IL-17 signaling, and Th17 cell differentiation (presented as red underbars). Pathways exhibiting significant changes (FDR < 0.05) were identified. The size of the spot represents the number of genes, and the color represents the FDR value. The vertical coordinates represent the KEGG pathways with significant enrichment, whereas the horizontal coordinates represent the rich factor.

B–C. Gene ontology (GO) terms for the intersection targets of the PPI network topology analysis for the GSE46750, GSE51588, and GSE29746 profiles. C. Biological process and D. Cellular component. Top 20 GO functional categories with FDR < 0.05 are presented.

D. The 12 pathways located at the intersection of GSE46750, GSE51588, and GSE29746 in terms of KEGG enrichment analysis containing 3 inflammation-related signaling pathways: IL-17, TNF, and NF-κB signaling, and 9 other pathways: AGE-RAGE signaling pathway in diabetic complications, Kaposi sarcoma-associated herpesvirus infection, hepatitis B, fluid shear stress and atherosclerosis, human T-cell leukemia virus 1 infection, endocrine resistance, bladder cancer, p53 signaling pathway, and transcriptional misregulation in cancer.

**Table S1. Ingredients of Bushen Zhuangjin Decoction**

| **Chinese name** | **Pinyin**  **(Chinese phonetic alphabet）** | **English name** | **Scientific name** | **Part used** | **Proportion of ingredients (100%)** |
| --- | --- | --- | --- | --- | --- |
| 熟地黄 | Shu Di Huang | Radix Rehmanniae Preparata (RRP) | Rehmannia glutinosa (Gaertn.) DC. | Prepared Root | 11.43% |
| 当 归 | Dang Gui | Radix Angelicae Sinensis (RAS) | Angelica sinensis (Oliv.) Diels | Dried Root | 11.43% |
| 续 断 | Xu Duan | Radix Dipsaci (RD) | Dipsacus asperoides C.Y.Cheng & T.M.Ai | Dried Root | 11.43% |
| 牛 膝 | Niu Xi | Radix Achyranthis Bidentatae (RAB) | Radix Achyranthis Bidentatae | Dried Root | 9.52% |
| 茯 苓 | Fu Ling | Poria (P) | Smilax glabra Roxb | Dried Sclerotium | 11.43% |
| 青 皮 | Qing Pi | Pericarpium Citri Reticulatae Viride (PCRV) | Citrus × aurantium L | Dried  Peel | 4.76% |
| 山茱萸 | Shan Zhu Yu | Fructus Corni (FC) | Cornus officinalis Siebold & Zucc | Dried Fruit | 11.43% |
| 杜 仲 | Du Zhong | Cortex Eucommiae (CE) | Eucommia ulmoides Oliv | Dried Bark | 9.52% |
| 白 芍 | Bai Shao | Radix Paeoniae Alba (RPA) | Paeonia lactiflora Pall | Dried Root | 9.52% |
| 五加皮 | Wu Jia Pi | Cortex Acanthopanax Radicis (CAR) | Eleutherococcus nodiflorus (Dunn) S.Y.Hu | Dried Root Bark | 9.52% |
