# Supplementary material for: Identification of the Key Role of NF-κB Signaling Pathway in the Treatment of Osteoarthritis With Bushen Zhuangjin Decoction, a Verification Based on Network Pharmacology Approach
Source: Front Pharmacol. 2021 Apr 12;12:637273. doi: 10.3389/fphar.2021.637273 (PMC8072665; doi:10.3389/fphar.2021.637273)
Supplement: Supplementary file 1 [file datasheet1.docx]

Dear Editor

We provided raw data of the western blots for all replicates that contributed to the quantitative analysis.

**Fig. 2. The effects of LPS and BZD exposure on protein expression.**

Three groups were performed in one film, from left to right was Control, KOA and KOA+BZD respectively.

**1. TNF-α**

Round 1 (shown in Fig. 2A)

Round 2

Round 3

**2. IL-1β**

Round 1

Round 2(shown in Fig. 2A)

Round 3

3. **Myd88**

Round 1

Round 2(shown in Fig. 2A)

Round 3

4. **MMP-3**

Round 1(shown in Fig. 2A)

Round 2

Round 3

5. **TLR4**

Round 1

Round 2

Round 3(shown in Fig. 2A)

**6. NF-κB P65**

Round 1

Round 2(shown in Fig. 2A)

Round 3

**7. GAPDH**

Round 1(shown in Fig. 2A)

Round 2

Round 3

**Fig. 9. Verification of the targeted NF-κB signaling pathway**

Four groups were performed in one film, from left to right was Control, KOA, KOA+BZD and KOA+PDTC respectively.

**1. NF-κB P65**

Round 1

Round 2(shown in Fig. 2A)

Round 3

**2. IKK-α**

Round 1(shown in Fig. 2A)

Round 2

Round 3

**3. IKK-β**

Round 1

Round 2

Round 3(shown in Fig. 2A)

**4. MMP-3**

Round 1

Round 2(shown in Fig. 2A)

Round 3

**5. Collagen II**

Round 1(shown in Fig. 2A)

Round 2

Round 3

**6. GAPDH**

Round 1

Round 2(shown in Fig. 2A)

Round 3

Best wishes

Sincerely

Tetsuya Asakawa, M.D., Ph.D.

Department of Neurosurgery, Hamamatsu University School of Medicine

Handayama, 1-20-1, Higashi-ku, Hamamatsu-city,

Shizuoka, 431-3192, Japan

Tel: 81-53-435-2283

Fax: 81-53-435-2282

E-mail: asakawat1971@gmail.com
